# Mesenchymal stem cell-derived exosomal miR-27b-3p alleviates liver fibrosis via downregulating YAP/LOXL2 pathway

**DOI:** 10.1186/s12951-023-01942-y

**Published:** 2023-06-16

**Authors:** Fang Cheng, Fuji Yang, Yanjin Wang, Jing Zhou, Hui Qian, Yongmin Yan

**Affiliations:** 1 Department of Laboratory Medicine, Wujin Hospital Affiliated with Jiangsu University, Jiangsu University, Changzhou, 213017 China; 2 Key Laboratory of Laboratory Medicine of Jiangsu Province, School of Medicine, Jiangsu University, Zhenjiang, 212001 China; 3 Changzhou Key Laboratory of Molecular Diagnostics and Precision Cancer Medicine, Wujin Hospital Affiliated with Jiangsu University, Changzhou, 213017 China; 4 Wujin Institute of Molecular Diagnostics and Precision Cancer Medicine of Jiangsu University (Wujin Clinical College of Xuzhou Medical University), Changzhou, 213017 China

**Keywords:** MSC, Exosome, miR-27b-3p, LOXL2, YAP, Liver fibrosis, Collagen crosslinking

## Abstract

Lysyl oxidase-like 2 (LOXL2) is an extracellular copper-dependent enzyme that plays a central role in fibrosis by catalyzing the crosslinking and deposition of collagen. Therapeutic LOXL2 inhibition has been shown to suppress liver fibrosis progression and promote its reversal. This study investigates the efficacy and underlying mechanisms of human umbilical cord-derived exosomes (MSC-ex) in LOXL2 inhibition of liver fibrosis. MSC-ex, nonselective LOX inhibitor β-aminopropionitrile (BAPN), or PBS were administered into carbon tetrachloride (CCl4)-induced fibrotic livers. Serum LOXL2 and collagen crosslinking were assessed histologically and biochemically. MSC-ex’s mechanisms on LOXL2 regulation were investigated in human hepatic stellate cell line LX-2. We found that systemic administration of MSC-ex significantly reduced LOXL2 expression and collagen crosslinking, delaying the progression of CCl4-induced liver fibrosis. Mechanically, RNA-sequencing and fluorescence in situ hybridization (FISH) indicated that miR-27b-3p was enriched in MSC-ex and exosomal miR-27b-3p repressed Yes-associated protein (YAP) expression by targeting its 3ʹ untranslated region in LX-2. LOXL2 was identified as a novel downstream target gene of YAP, and YAP bound to the LOXL2 promoter to positively regulate transcription. Additionally, the miR-27b-3p inhibitor abrogated the anti-LOXL2 abilities of MSC-ex and diminished the antifibrotic efficacy. miR-27b-3p overexpression promoted MSC-ex mediated YAP/LOXL2 inhibition. Thus, MSC-ex may suppress LOXL2 expression through exosomal miR-27b-3p mediated YAP down-regulation. The findings here may improve our understanding of MSC-ex in liver fibrosis alleviation and provide new opportunities for clinical treatment.

**Supplementary Information:**

The online version contains supplementary material available at 10.1186/s12951-023-01942-y.

## Introduction

Liver fibrosis develops in response to various chronic injuries such as viral infection, nonalcoholic steatohepatitis (NASH), nonalcoholic fatty liver disease (NAFLD), alcohol-related fatty liver diseases (AFLD), and autoimmune hepatitis. It can give rise to cirrhosis or hepatocellular carcinoma (HCC) and has become one of the leading causes of death, accounting for approximately 2 million deaths per year worldwide [1, 2]. However, curative treatment is unavailable for clinical use, and liver transplantation is the only practical option for decompensated cirrhosis or HCC [3]. Collagen crosslinking is essential to liver fibrosis progression and limiting its reversibility. The development of effective therapeutics that can reverse collagen crosslinking is urgently required.

The Lysyl oxidase (LOX) family (LOX and LOXL1–4) are essential enzymes that promote crosslinking of collagens by oxidatively deaminating lysine residues. Like all LOX members, LOXL2 is secreted to the extracellular matrix (ECM), where they participate in crosslinking of fibrillar collagen, a significant component of fibrosis. The distinct role of LOXL2 in a wide range of diseases, including liver fibrosis, has been elaborated [4–7]. Recent studies suggest that targeting LOX, LOXL1, or LOXL2 is an attractive strategy for treating mouse liver fibrosis [8–10]. However, selective LOXL2-blocking monoclonal antibodies failed to treat fibrosis of multiple organs, especially liver fibrosis [11–14]. The lack of potency of this allosteric LOXL2 antibody and insufficient target engagement within the fibrotic scar is likely causes [15]. Thus, exploring potential mechanisms associated with LOXL2 regulation and developing novel agents targeting LOXL2 is essential for antifibrotic therapy.

Recently, MSC transplantation has emerged as an attractive therapeutic for treating liver fibrosis and cirrhosis [16]. MSC-based clinical trials are being investigated for alcoholic liver cirrhosis and liver fibrosis. Despite their beneficial effects in repairing the liver injury, the application of MSC is limited by malignant transformation, tumor-associated fibroblasts (TAF) transition, or risk of low engraftment in vivo [17, 18]. Recently, most studies have shown that the therapeutic potential of MSC in liver diseases can be attributed to exosomes [19]. Exosomes are nanosized extracellular membrane vesicles that contain functional nucleic acids (mRNAs and microRNAs), proteins, and lipids and function as mediators of intercellular communication. MSC-derived exosomes (MSC-ex) have been suggested as a safe and effective cell-free based nanomaterial for liver injury [19]. They have similar reparative properties as their cellular counterparts in liver injury repair [20]. We previously demonstrated that human umbilical cord MSC-ex could promote the recovery of hepatic oxidant injury and alleviate liver fibrosis [21, 22]. However, mechanisms underlying the effects of MSC-ex transplantation on liver fibrosis were unclear. It is also unknown whether MSC-ex could regulate LOXL2 expression and collagen crosslinking to suppress the progression of liver fibrosis.

In this study, we investigated the effects and molecular mechanisms of MSC-ex on LOXL2 modulation in carbon tetrachloride-induced liver fibrosis mouse models and in vitro cell culture systems. Hepatic stellate cells (HSC) are the primary cellular sources of LOXL2 in fibrotic livers. MSC-ex efficiently inhibits LOXL2 secretion of activated HSC and collagen crosslinking and suppresses liver fibrosis progression. We also demonstrate that LOXL2 can be transcriptionally regulated by Yes-associated protein (YAP), which was overexpressed in TGFβ activated HSC. In addition, MSC-ex-derived miR-27b-3p promoted YAP down-regulation, reducing LOXL2 expression, and inhibited collagen crosslinking and fibrosis progression in vivo. These findings provide a better understanding of using MSC-ex for LOXL2 inhibition and liver fibrosis therapy.

## Materials and methods

### Cell culture

The human umbilical cord was gained from informed, healthy parturients, and MSC was isolated from the human umbilical cord and identified as described previously [22]. All clinical procedures followed the protocols approved by the ethics committee of Jiangsu University, and the approved guidelines carried out the methods. All participants have written consent for the present study. MSC was cultured in L-DMEM (Gibco, Thermo Fisher Scientific) containing 10% fetal bovine serum (FBS) (Bovogen, Australia). Human immortalized L02 cells (Chinese Academy of Sciences) were maintained in RPMI 1640 containing 10% FBS (Bovogen, Australia). HEK293T cells (ATCC) were maintained in L-DMEM (Gibco, Thermo Fisher Scientific) containing 10% fetal bovine serum (FBS) (Bovogen, Australia). Human immortalized HSC cell line LX-2 (Chinese Academy of Sciences) was maintained in H-DMEM (Gibco) containing 10% FBS (Bovogen, Australia). All cells were cultured at 37 °C with 5% CO_2_ and tested for mycoplasma contamination.

### Isolation and characterization of MSC-ex

MSC-ex was isolated and purified as our previously established method [21]. MSC was cultured in an FBS-free medium, in which bovine exosomes and protein aggregates were removed by ultra-centrifugation at 100,000×*g* for 16 h at 4 °C. 500 mL condition medium from MSC at passages 3 to 6 was collected, and centrifuged at 2000×*g* for 20 min to remove cell debris. Then, Supernatants were concentrated using a 100 KDa molecular weight cut-off (MWCO) ultrafiltration filter per the manufacturer’s instructions (Millipore, USA). After filtration with 0.22 μM filter membrane, the exosomes-enriched fraction was transferred to a 15 mL sterile centrifuge tube. MSC-ex was precipitated from the concentrates using ExoQuick-TC extracellular vesicle (EV) isolation Kit following the protocol (System Biosciences, USA). The protein concentration of the extracted exosomes was quantified by a BCA protein assay kit (Pierce, ThermoFisher). The morphology of MSC-ex was observed by transmission electron microscopy (FEI Tecnai 12, Philips). The amount and size distribution of MSC-ex was measured by NanoSight tracking analysis (NTA) with NTA 3.1 Software (NanoSight, Malvern, UK).

### CCl4-induced mouse model of liver fibrosis and MSC-ex injection

All experiments involving animals were conducted according to the ethical policies and procedures approved by the Jiangsu University ethics committee (Approval no. UJS-IACUC-AP-2020033127). BALB/c female mice, 4–5 weeks old, were treated with carbon tetrachloride (CCl4) (10%) for 6 weeks to induce liver fibrosis as described. To analyze the effect of MSC-ex on LOXL2 expression and liver fibrosis, mice were randomized into four groups: PBS group, mice injected with 1 mL PBS (*n* = 6); 3-aminopropionitrile fumarate salt (BAPN) group, mice treated with BAPN (125 mg/kg, Sigma-Aldrich, *n* = 6) once per day; and MSC-ex 12.5 mg/kg body weight (*n* = 6), 25 mg/kg body weight (*n* = 6) groups, mice treated with MSC-ex twice a week. PBS, BAPN, and MSC-ex were administered by tail vein. At 4 weeks post MSC-ex injection, mice were sacrificed to collect blood and liver samples for further analysis.

### MSC-ex labeling and tracking in mice and LX-2 Cells

MSC-ex was incubated with DiR (DiIC 18(7); 1,1′-dioctadecyl-3,3,3′,3′-tetramethylindotricarbocyanine iodide) (UElandy Biology, China) or PKH67 (Sigma-Aldrich, USA) for 30 min at 37 °C according to the manufacturer’s instructions. After washing with PBS, DiR or PKH67 labeled MSC-ex were concentrated with a 100 KDa molecular weight cut-off (MWCO) ultrafiltration filter at 1000×*g* for 30 min to remove the non-binding dye. For in vivo tracking of MSC-ex in mice, DiR labeled MSC-ex were injected intravenously and analyzed using a Maestro In Vivo Imaging System (CRI, MA, USA). In vivo, spectral imaging from 690–850 nm was performed using an exposure time of 150 ms per image frame. For the distribution of MSC-ex in LX-2, PKH67 labeled MSC-ex (PKH67-ex) was incubated with LX-2 cells at 37 °C for 24 h and observed with confocal microscopy.

### Western blot

Whole-cell or MSC-ex lysates were prepared in RIPA lysis buffer (Beyotime, Shanghai, China). Protein concentration was determined using the BCA assay kit (Vazyme Biotech, Nanjing, China). Equal amounts of lysates were loaded and separated on a 10% or 12% SDS-PAGE gel. Standard Western blot used primary antibodies and a peroxidase-linked, species-specific, anti-mouse, anti-rat, or anti-rabbit IgG (CWBIO, China). The following primary antibodies were used: CD9 (1:500, Bioworld, USA, BS3022), CD63 (1:1000, Abcam, UK, ab271286), Calnexin (1:2000, Sigma-Aldrich, USA, BS1438), TSG101 (1:1000; Abcam, UK, BS91381), α-SMA (1:1000; Bioworld, USA, BM0002), LOXL2 (1:2000; Bioworld, USA, MB63843), YAP (1:1000; Bioworld, USA, BS2000), β-actin (1:2000; ABclonal, UK, Rabbit, AC006). and GAPDH (1:2000; Abclonal, China, AC001). Proteins were detected with an ECL detection system (Amersham Pharmacia Biotech, Little Chalfont, UK). Western blot results were quantitated using ImageJ software; protein expression was normalized to GAPDH.

### Quantitative reverse transcription PCR

The total RNA of LX-2 cells and mouse livers were extracted with Trizol according to the manufacturer’s instructions (Invitrogen). 1 µg of total RNA was used for the reverse transcription of RNA into cDNA in a reaction using the SuperScriptTM II RT kit according to the manufacturer’s instructions (Invitrogen). SYBR-Green I-based Real-Time PCR kit (Vazyme Biotech Co., Ltd, China) was used, and relative gene expression quantitation was determined using the 2^−ΔΔCT^ method and normalized to the β-actin gene. The PCR primers are listed in Table 1 (Shanghai Bio-Engineering, China). The fluorescence signals were detected by CFX96 Touch™ Real-Time PCR Detection System (Bio-Rad, USA). MiScript primer assays were used for the semiquantitative determination of human miR-27b-3p (Qiagen GmbH, Germany). Relative gene expression normalized to U6 was calculated using the 2^−ΔΔCt^ method. There were three or six replicates per group.

**Table 1:** Primers for real-time quantitative PCR

Genes	Primer sequence (5′-3′)	Annealing temperature (°C)	Product size (bp)
Human LOXL2	For: CTGCAAGTTCAATGCCGAGT	60	149
	Rev: TCTCCACCAGCACCTCCACTC		
Human Col1A2	For: CTACTGGTGCCAGAGGACTT	58	138
	Rev: TAGGGCCTCTCTTTCCTTCT		
Mouse LOXL2	For: TTCTGCCTGGAGGACACTGAGT	58	139
	Rev: TTCTGCCTGGAGGACACTGAGT		
Mouse/Human β-actin	For: CACGAAACTACCTTCAACTCC	56	265
	Rev: CATACTCCTGCTTGCTGATC		
LOXL2 ChIP	For: GGTTTGTCTCCTCAGGGAGTG	57	102
	Rev: GCGAGCTGCAAAACAAGGGA		

### Serum assay

Serum LOXL2 levels in fibrotic mice treated with MSC-ex or BAPN were determined using an ELISA (Enzyme-linked Immunosorbent Assay) kit (XinYu Biotech, Shanghai, China) according to the manufacturer’s instructions. Serum alanine aminotransferase (ALT), aspartate aminotransferase (AST), lactate dehydrogenase (LDH), and UREA were measured with an automated biochemical analyzer.

### Immunohistochemistry and immunofluorescence of liver tissues

LOXL2, YAP, and α-SMA protein expressions were analyzed using MSC-ex or BAPN-treated mouse tissue samples. Mouse tissue sections (4 µm thick) of formalin-fixed, paraffin-embedded liver specimens were deparaffinized in xylene and rehydrated in graded alcohol. Standard immunohistochemical procedures were performed on liver tissue sections using anti-LOXL2 (1:50; Santa Cruz Biotechnology, USA), anti-YAP (1:50; Bioworld, USA), or anti-α-SMA antibody (1:50; Bioworld, USA). Signals were visualized using 3, 3′-Diamino-benzidine tetrahydrochloride (Boster Biology, Wuhan, China). A brown membrane, cytoplasmic, and nuclear staining indicated a positive reaction according to different markers. The staining result of LOXL2, YAP, and α-SMA expression was determined by the percentage of positive cells by two investigators blinded to the data. Immunofluorescence staining was performed using anti-CD9 (1:100, Bioworld, USA), anti-LOXL2 (1:50), anti-YAP (1:50), or anti-α-SMA antibody (1:50). Negative controls with isotype IgG were run in parallel. Images were acquired using a laser scanning confocal microscope (Nikon, Tokyo, Japan).

### Hematoxylin and eosin (HE) staining

Formalin-fixed paraffin-embedded cardiac, liver, spleen, lung, and kidney tissues sections were stained with Masson Trichrome (MT) (Gefan, China) and Sirius Red (Chondrex, USA) according to the instruction of the manufacturer. To analyze hepatic collagen distribution, 10 fibrotic septa randomly selected from the right and left liver lobes of 6 individual mice/groups were assessed. Collagen extent was expressed as a percentage of stained area in each liver section.

### TGFβ induced LX-2 activation and MSC-ex treatment

LX-2 was cultured in a 6-well plate until it reached approximately 50–60% confluence. Then cells were randomized into four groups: PBS, LX-2 treated with PBS, TGFβ: LX-2 treated with 10 ng/mL TGFβ, TGFβ/MSC-ex, LX-2 treated with 10 ng/mL TGFβ and 100 or 200 μg/mL MSC-ex, TGFβ/BAPN: LX-2 treated with 10 ng/mL TGFβ and 1.0 mg/mL BAPN. These cells were treated for 48 h for further investigation.

### Immunofluorescence of LX-2

LX-2 cells were fixed in 4% paraformaldehyde for 10 min and permeabilized with 0.1% Triton X-100 for 10 min at room temperature. For examining LOXL2, YAP, and α-SMA protein levels, LX-2 cells were blocked with 5% BSA and incubated with anti-LOXL2, anti-YAP, or anti-α-SMA primary antibody at 4 °C overnight; after incubation and washing, fluorescent-labeled secondary antibody was added with the necessary incubation and washing. Then the slides were counterstained with 4′,6-diamidino-2-phenylindole (DAPI) for nuclear staining and examined under a laser scanning confocal microscope (Nikon, Tokyo, Japan).

### Adenoviral overexpression and knockdown of YAP

Adenoviruses expressing full-length human YAP (Ad-YAP), GFP alone (Ad-GFP), YAP shRNA (sh-YAP), and control shRNA (sh-Ctr) were constructed. Full-length human YAP cDNA was inserted into pAV(Exp)-CMV > YAP/HA-IRES-Egfp adenoviral vectors to generate Ad-YAP expression vectors. The adenoviral YAP shRNA vector was packaged by the vector ADV1(U6/CMV-GFP) with YAP shRNA oligonucleotides. The shRNA oligonucleotide sequences are listed in Table 2. Recombinant adenovirus was produced by co-transfecting 293A cells, as described previously. YAP overexpression and knockdown efficiency were evaluated using quantitative Reverse Transcription PCR and western blot. To prepare YAP-modified 293T and LX-2 cells, Ad-YAP and sh-YAP transfected cells were collected.

**Table 2:** shRNA oligonucleotides

Genes	Sequence (5′-3′)
sh-YAP	CCGGGCCACCAAGCTAGATAAAGAACTCGAGTTCTTTATCTAGCTTGGTGGCTTTTTG
sh-Ctr	CCGGGCAAGCTGACCCTGAAGTTCATCTCGAGATGAACTTCAGGGTCACGTTGCTTTTTG

### Promoter activity analysis

The LOXL2 promoter constructs pcDNA3.1-LOXL2-907 (pLOXL2-907), pcDNA3.1-LOXL2-826 (pLOXL2-826) and pcDNA3.1-LOXL2-328 (pLOXL2-328) were Chemically synthesized (GENERAL BIOL, Anhui, China). For the LOXL2 reporter assay, HEK293T and LX-2 cells were seeded in 24-well plates. The LOXL2 promoter constructs and Renilla luciferase reporter were cotransfected with plasmid DNA of pcDNA3.1-vector (p3.1), pcDNA3.1-YAP (pYAP), and in some experiments, control shRNA (sh-Ctr) or YAP shRNA (sh-YAP). Lipofectamine 2000 reagent (Life Technologies) was used according to the manufacturer’s instructions. Both firefly and Renilla luciferase activity were measured using a dual-luciferase assay system (Promega, Madison, USA) 48 h after transfection. The LOXL2 promoter activity was normalized with the Renilla luciferase activity. Three biological repeats were used for each sample in the dual luciferase reporter.

### Chromatin immunoprecipitation quantitative real-time PCR (ChIP-qPCR)

Chromatin immunoprecipitation (ChIP) assay followed the manufacturer’s instructions (Millipore, Billerica, MA). Briefly, formaldehyde was used to cross-link proteins with DNA, and 2 × 10^7^ 293T or LX-2 cells were lysed in sodium dodecyl sulfate lysis buffer. The cell lysate was sonicated to shear the DNA to 400- to 600-bp lengths. Chromatin samples were then precleared with a salmon sperm DNA/protein A agarose 50% slurry for 30 min at 4 °C and immunoprecipitated overnight with anti-IgG or anti-YAP (Bioworld, USA, BS9920M). The purified DNA fragments were subjected to quantitative PCR using primers listed in Table 1. Four biological replications were included in each treatment.

### Luciferase reporter assays

YAP was predicted as a target of miR-27b-3p by TargetScan (http://targetscan.org/). The 3ʹ‑untranslated region (UTR) of the human YAP gene containing either wild-type (WT) or mutant-type (MT) binding sites of miR-27b-3p were synthesized and inserted in the pGL3 vector downstream of the firefly luciferase gene to form pGL3-YAP, named as YAP-WT and YAP-Mut. LX-2 cells (2.5 × 10^4^/well) were seeded in 24-well plates and were co-transfected with miR-27b-3p mimics or negative control (NC) miRNA mimics and control reporter plasmids pGL3, YAP-WT or YAP-Mut using Lipofectamine 2000. The Dual-Luciferase Reporter assay system (Promega, USA) was applied to examine the activities of Renilla and firefly luciferase based on the manufacturer’s protocols at 24 h post-transfection. Firefly luciferase activity was normalized to Renilla luciferase activity.

### Crystal droplet digital PCR™ assays

Droplet digital PCR was performed using the naica^®^ system (Stilla Technologies, France). 125 ng RNA was isolated from MSC-ex (10^4^ particles). 500 ng RNA in 20 μL was used per reaction to generate cDNA by reverse transcription. The reaction mixture consisted of 2 μL QX200 ddPCR EvaGreen (Bio-Rad), 1 μL Laxa Fluor 647 dye (Thermo Fisher), 3.75 μL ddPCR™ supermix for probes (Bio-Rad), 1 μL primer, and 5 μL DNA template. Finally, RNase-free H_2_O was added to make a total reaction volume of 25 μL. The two-step method is used for ddPCR, and the cycling procedure consists of a pre-denaturation step at 95 °C for 5 min, followed by 45 cycles of denaturation at 95 °C for 10 s, annealing at 62 °C for 45 s, and 72 °C for 30 s. After the PCR cycle, the sapphire chip was transferred to a Naica Prism 3 fluorescence reader for imaging. Crystal Miner software (Stilla Technologies) was used for data analysis.

### Fluorescence in situ hybridization (FISH)

Cy3-labeled miR-27b-3p probe sequence was purchased from GenePharma, China. 3 × 10^5^ cells were cultured in 24-well glass slide plates. 4% paraformaldehyde was used to fix the cells. 15 min of TRITON X-100 treatment was followed by incubation with blocking solution for 30 min (37 °C). Cy3-labeled miR-27b-3p fluorescent probe solution was incubated with cells in an in situ hybridizer for 14 h (37 °C). Tween 20 was used to wash the cells. For liver tissue, 7 mm frozen liver tissue sections were digested with proteinase K and incubated in a blocking buffer for 30 min (37 °C). Prepare the cy3-labeled miR-27b-3p fluorescent probe working solution at a volume ratio of 1:1. Incubate with liver tissue slices for 14 h (37 °C) in an in situ hybridization instrument. Wash sections with deionized formamide at 43 °C to denature unhybridized probes. Sections were washed three times with sodium citrate buffer (60 °C). FISH images were then captured by confocal microscopy.

### miR-27b-3p mimics or inhibitors transfection

Human miR-27b-3p mimics, negative control mimics, and miR-27b-3p inhibitors, negative control inhibitors were purchased from GenePharma, China. According to the instruction of the manufacturer, miR-27b-3p mimics (25 nM, 50 nM) and miR-27b-3p inhibitors (50 nM, 100 nM) were transiently transfected into LX-2 cells using Lipofectamine 2000 in Opti-MEM™ medium (Invitrogen, USA) at 70–80% confluency in 6-well culture plates. At 4–6 h post-transfection, the culture medium was replaced with MEM with 10% FBS for another 48 h. The transfected cells were collected for further investigation.

### miR-27b-3p knockdown of MSC-ex

Negative control inhibitors, miR-27b-3p inhibitors (50 nM, 100 nM), were transiently transfected into MSC using Lipofectamine 2000 in Opti-MEM™ medium (Invitrogen, USA) at 70–80% confluency in 6-well culture plates. At 4–6 h post-transfection, the culture medium was replaced with an FBS-free medium for another 48 h. Then total RNA of inhibitors transfected MSC was collected for miR-27b-3p quantification. miR-27b-3p inhibitors transfected MSC-ex (miR-27b^in^-ex), or negative control inhibitors transfected MSC-ex (NC^in^-ex) were isolated, purified, and washed as our previously established method. After concentration and structure identification, miR-27b^in^-ex or NC^in^-ex were stored at − 70 °C for further use. Then LX-2 cells were treated with NC^in^-ex (200 μg/mL) or miR-27b^in^-ex (2000 μg/mL) for 48 h and collected for further investigation.

### miR-27b-3p mimics the loading of exosomes

miR-27b-3p mimics were passively loaded into MSC-ex by the sonication method. MSC-ex was mixed with 50 nM miR-27b-3p mimics or negative control mimics and sonicated at 500 v, 2 kHz, 10% power, 6 cycles by 4 s pulse/2 s pause, cooled down on the ice for 2 min, and then sonicated again using Qsonica Sonicator Q700 (Misonix, USA). Then miR-27b-3p overexpressed MSC-ex (miR-27b^oe^-ex) or negative control mimics overexpressed MSC-ex (NC^oe^-ex) were washed with PBS 3 times to remove residual miRNA. After concentration and structure identification, miR-27b^oe^-ex or NC^oe^-ex were stored at − 70 °C for further use. Then LX-2 cells were treated with NC^oe^-ex (50 μg/mL) or miR-27b^oe^-ex (50 μg/mL) for 48 h and collected for further investigation.

### Statistical analysis

Statistical analyses were performed using GraphPad Prism version 8.3.0 (San Diego, CA, USA). All the data is presented as mean ± SD. The Student’s t-test was used for comparisons between the two groups. One-way analysis of variance (*ANOVA*) followed by Dunnett was used for studies involving more than two groups. A two-sided p < .05 was considered statistically significant.

## Results

### MSC-ex inhibited LOXL2 expression and collagen crosslinking in CCl4-induced liver fibrosis

**Fig. 1:**
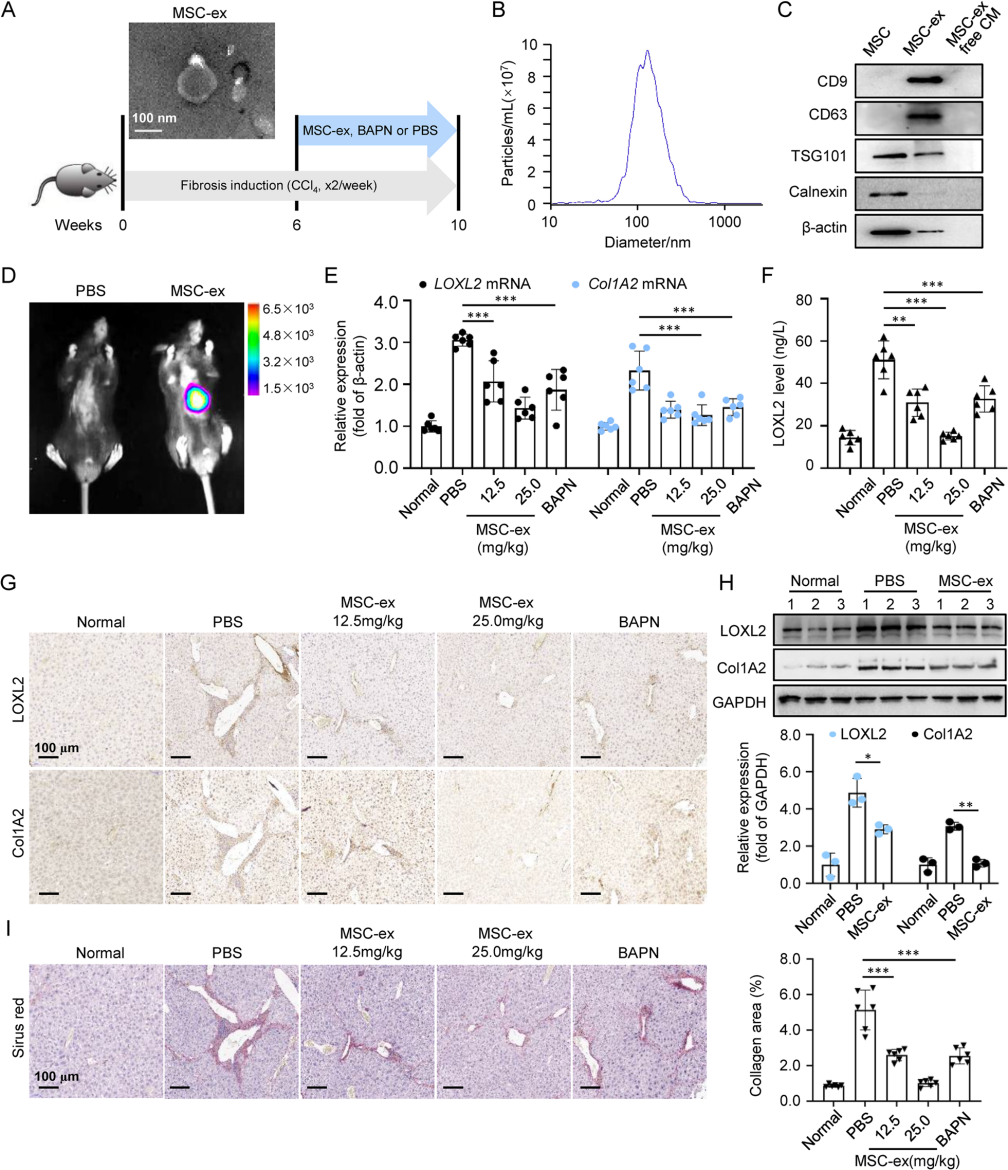
MSC-ex homing to the fibrotic liver and inhibited LOXL2 expression. **A** TEM image of MSC-ex and schematic illustration of in vivo experiments. Briefly, mice were administered with MSC-ex (50 μg or 100 μg), BAPN, or PBS at 6 weeks to 10 weeks on CCl4-induced liver fibrosis (n = 6). Scale bars, 100 nm. **B** Nanoparticle tracking analysis of MSC-ex. **C** Western blot analysis of exosomal markers CD9, CD63, Tsg101, and ER membrane marker Calnexin in MSC-ex. **D** Imaging of fluorescence intensity in mice from PBS or DiR labeled MSC-ex group at 24 h post-treatment. Scale bars, 1.0 cm. **E** qRT-PCR analysis for LOXL2 and Col1A2 mRNA in livers from PBS, MSC-ex, or BAPN-treated mice (n = 6; ***p < .001). **F** ELISA analysis for serum LOXL2 in PBS, MSC-ex, or BAPN-treated mice (n = 6; **p < .01, ***p < .001). **G** Immunohistochemistry staining for LOXL2 and Col1A2 in mouse livers. Scale bars, 100 μm. **H** Western blot analysis and quantification for LOXL2 and Col1A2 protein in mouse livers (n = 3; **p < .01). **I** Sirus Red staining of collagen deposition in mouse livers. The collagen area of fibrotic livers was quantified (n = 6; ***p < .001). Scale bars, 100 μm

To evaluate the effect of MSC-ex on LOXL2 expression, progressive liver fibrosis was induced in BALB/c mice by repeated carbon tetrachloride (CCl4) injections for up to 6 weeks. We administered MSC-ex treatment concurrently with fibrosis induction for another 4 weeks. PBS or pan-Lox inhibitor BAPN was administered in parallel (Fig. 1A). Surface markers, morphology, and size characterized MSC-ex isolated from human umbilical cord MSC-conditioned medium. The round spherical shape with a diameter of 30–100 nm of MSC-ex was observed by transmission electron microscopy (TEM) and nanoparticle tracking analysis (NTA) (Fig. 1A, B). Western blot also confirmed that MSC-ex was positive for exosome markers such as CD9, CD63, and TSG101, whereas endoplasmic reticulum membrane marker Calnexin was undetected (Fig. 1C).

The biodistribution of MSC-ex was investigated by labeling exosomes with DiR. In vivo, fluorescent imaging showed that DiR-labeled MSC-ex administered by tail vein targeted injured livers at 24 h post-injection (Fig. 1D, Additional file 1: Figure S1). qRT-PCR revealed that MSC-ex and BAPN, but not PBS, decreased LOXL2 and Col1A2 mRNA expression within 4 weeks of treatment (Fig. 1E). Serum LOXL2 was also reduced in animals given 12.5 mg/kg and 25 mg/kg MSC-ex at 4 weeks post-injection (Fig. 1F). As shown in Fig. 1G, immunohistochemistry revealed that LOXL2 expression was strongly induced after 10 weeks of CCl4. LOXL2 immunoreactivity was observed along the fibrotic septa in a pattern like the distribution of Col1A2. Treatment with 25 mg/kg MSC-ex significantly reduced LOXL2 and Col1A2 expression in the portal area. A similar reduction in LOXL2 was apparent in livers from the 25 mg/kg MSC-ex group compared with the BAPN group. Consistently, western blot quantitative analysis showed that both LOXL2 and Col1A2 protein expression was inhibited by 25 mg/kg MSC-ex (Fig. 1H). We then performed connective tissue staining to assess whether MSC-ex directly affected collagen crosslinking and fibrotic matrix stabilization. Sirus red staining revealed reduced liver scarring in the 25 mg/kg MSC-ex treated group. In contrast, the collagen area was partly reduced in the 12.5 mg/kg MSC-ex and BAPN-treated group, respectively (Fig. 1I). HE staining also indicated that MSC-ex had no significant impact on cardiac, liver, spleen, lung, and kidney tissue structure in healthy mice compared to the PBS group (Additional file 2: Figure S2A). Additionally, we observed no significant differences in liver function indicators (ALT, alanine aminotransferase; AST, aspartate aminotransferase), cardiac function indicators (LDH, lactate dehydrogenase), and renal function indicators UREA between the MSC-ex and PBS groups (Additional file 2: Figure S2B). These results suggest that MSC-ex is biologically safe for use. These findings indicate that MSC-ex can inhibit LOXL2 expression and collagen deposition, delaying the progression of CCl4-induced liver fibrosis.

### MSC-ex diminishes LOXL2 expression derived from activated HSC

**Fig. 2:**
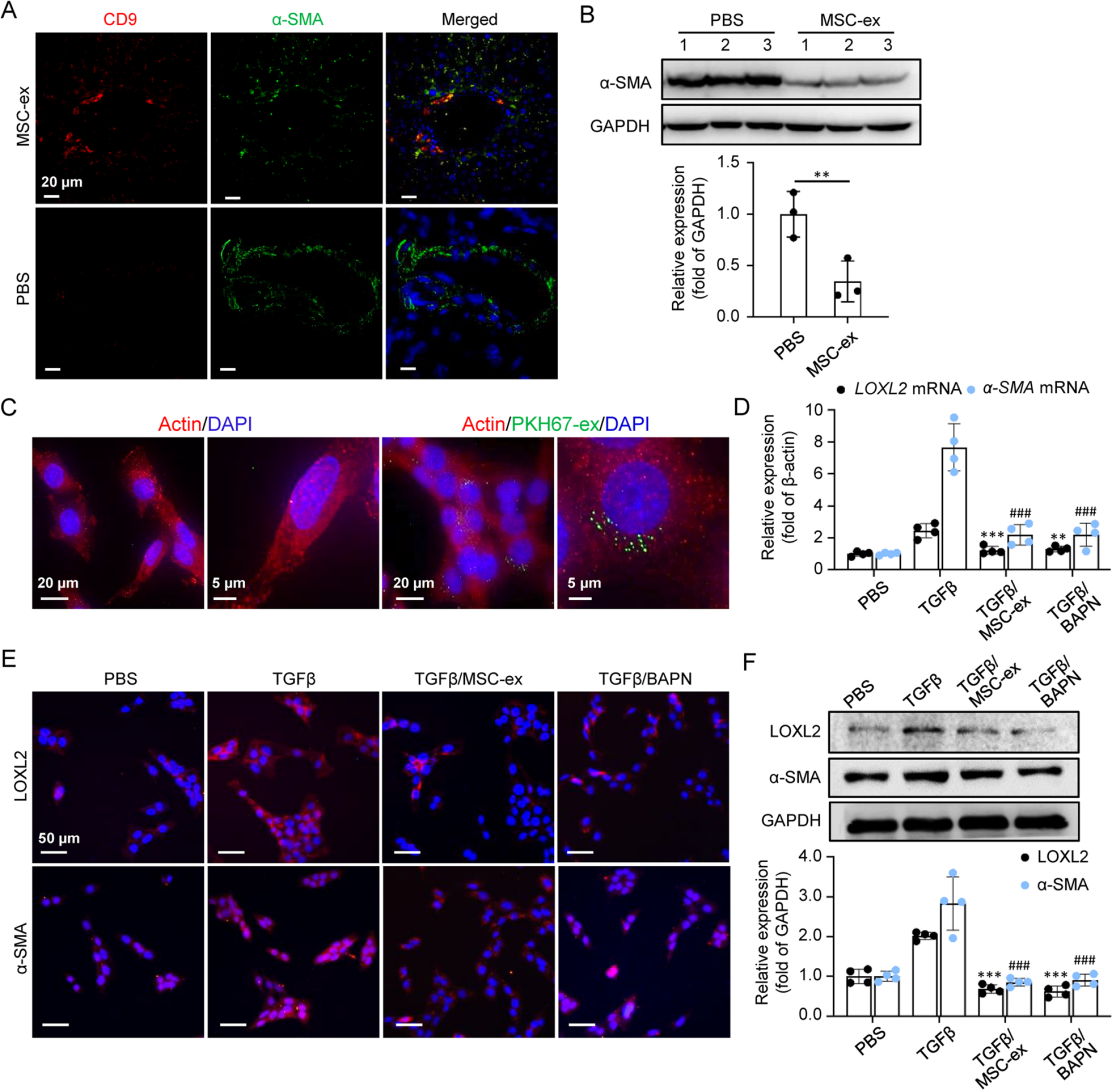
MSC-ex diminishes LOXL2 expression derived from activated HSC. **A** Immunofluorescence staining of exosomal marker CD9 (Red) and HSC activation marker α-SMA (Green) in PBS or MSC-ex treated mouse livers. Scale bars, 20 μm. **B** Western blot analysis and quantification of α-SMA in PBS or MSC-ex treated mouse livers (n = 3; **p < .01). **C** Representative fluorescent images showed the uptake of PKH67-labeled MSC-ex (PKH67-ex) by LX-2 cells. Scale bars, 20 μm. **D** qRT-PCR analysis for LOXL2 and α-SMA mRNA in PBS, MSC-ex, or BAPN treated LX-2 cells (n = 4; **p < .01, ***p < .001, and ^###^p < .001). **E** Immunofluorescence staining images of LOXL2 and α-SMA protein in PBS, MSC-ex (200 μg/mL), or BAPN (1.0 mg/mL) treated LX-2 cells. Scale bars, 50 μm. **F** Western blot analysis and quantification of LOXL2 and α-SMA protein in PBS, MSC-ex (200 μg/mL) or BAPN (1.0 mg/mL) treated LX-2 cells (n = 4; ***p < .001, ^###^p < .001)

Because activated hepatic stellate cells (HSC) are the primary source of collagen in established liver fibrosis, we sought to test whether HSC-activated myofibroblast functions as the cellular target of MSC-ex for anti-LOXL2 therapies. We also injected PKH26-labeled MSC-ex into liver fibrosis mice via tail vein injection. Immunofluorescence analysis showed that a small amount of PKH26-MSC-ex could localize in CD31^+^ endothelial cells, F4/80^+^ Kupffer cells, and albumin^+^ hepatocytes (Additional file 3: Figure S3). Double immunofluorescence for human exosome markers CD9 and activated HSC marker α-smooth muscle actin (α-SMA) in fibrotic livers revealed a 25 mg/kg MSC-ex location in the portoportal regions of the liver and a substantial reduction of α-SMA expression was apparent in MSC-ex located cells (Fig. 2A). Western blot confirmed a 77.4% reduction of α-SMA protein levels in MSC-ex-treated mice compared with PBS treatment (Fig. 2B). These data suggest a potential inhibition of MSC-ex on myofibroblast activation. To assess whether MSC-ex directly affected LOXL2 inhibition of activated HSC, LX-2 was incubated with PKH67 labeled MSC-ex (PKH67-ex). Confocal imaging showed that PKH67-ex mainly localized in the cytoplasm and around the nucleus after 12 h of incubation (Fig. 2C). These results implied that LX-2 could take MSC-ex in vitro. Then LX-2 was treated with TGFβ, TGFβ/MSC-ex, or TGFβ/BAPN for 24 h. QRT-PCR, immunofluorescence, and western blot analysis analyzed LOXL2 and α-SMA expression. Compared with the PBS group, LOXL2 and α-SMA mRNA and protein expression were significantly increased in TGFβ activated LX-2 (Fig. 2D–F). However, TGFβ induced LOXL2 and α-SMA expression was significantly downregulated by MSC-ex or BAPN (Fig. 2D–F). These results suggest that MSC-ex can inhibit LOXL2 expression in TGFβ activated HSC.

### YAP positively regulated the expression of LOXL2 at the transcriptional level

**Fig. 3:**
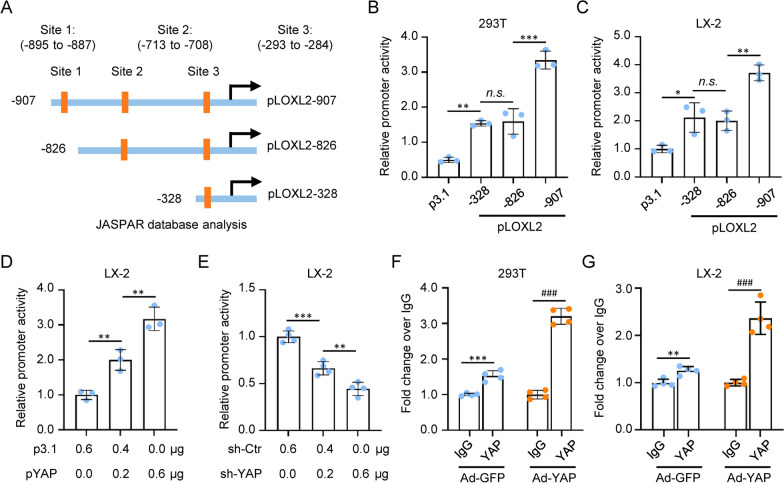
YAP positively regulated the expression of LOXL2 at the transcriptional level. **A** Schematic structure of the full-length LOXL2 promoter-reporter and its deletion-mutant constructs. Three potential YAP binding sites are indicated. **B**, **C** LOXL2 promoter activity in 293T and LX-2 cells at 48 h after transfection with pcDNA3.1-YAP (pYAP) and pcDNA3.1 (p3.1), pcDNA3.1-LOXL2 vector (pLOXL2) (n = 3; *p < .05, **p < .01, ***p < .001, and n.s., Not significant). **D** LOXL2 promoter activity in LX-2 cells cotransfected with pLOXL2-907 promoter construct and different doses of pYAP or p3.1 vector (n = 3; **p < .01). **E** LOXL2 promoter activity in LX-2 cells cotransfected with pLOXL2-907 promoter construct and different doses of YAP shRNA (sh-YAP) or control shRNA (sh-Ctr) vector (n = 4; **p < .01, ***p < .001). **F**, **G**. ChIP-qPCR analysis of YAP binding to the LOXL2 promoter region with potential YAP binding site in 293T or LX-2 cells with Ad-GFP or Ad-YAP transfection (n = 4; **p < .01, ***p < .001, and ^###^p < .001). The cell lysate was precipitated with an anti-IgG or anti-YAP antibody. qPCR with primers containing site 1 (− 895 to − 887) was used to identify the YAP binding

To elucidate the key molecule and potential signaling pathways involved in LOXL2 regulation, we analyzed the LOXL2 promoter using the UCSC genome website and JASPAR database. YAP was screened out as the transcriptional factor binding to the LOXL2 promoter region. Indeed, we identified three potential YAP-binding sites in the promoter region of LOXL2 by motif analysis (Fig. 3A). To validate the regulatory function of YAP on LOXL2 expression, LOXL2 promoter deletion constructs containing the region of sites 1, 2, and 3 were cotransfected with pcDNA3.1-YAP (pYAP) into 293T and LX-2 cells for LOXL2 promoter activity analysis, respectively. The results showed that the region from 293 to 284, 887 to 895 contains the response element(s) required for YAP regulatory control of LOXL2 promoter activity (Fig. 3B, C). In addition, a dose-dependent promotion effect was observed when the pLOXL2-907 vector was cotransfected with different doses of pcDNA3.1-YAP (pYAP)-expressing vector into LX-2 cells (Fig. 3D). In contrast, when YAP shRNA was used for cotransfection assay in LX-2 cells, LOXL2 promoter activity was drastically decreased (Fig. 3E). In the next step, we tested whether YAP directly binds to the LOXL2 promoter of 293T and LX-2 cells by chromatin immunoprecipitation (ChIP)-qPCR using YAP antibodies. As shown in Figures, YAP can directly bind to promoter sequences of LOXL2 while significantly lower binding was observed for control IgG (Fig. 3F, G). By elevated transcript levels, the binding of YAP to the promoter region was reinforced considerably in YAP-overexpressing 293T and LX-2 cells (Fig. 3F, G). These results demonstrate the YAP's direct transcriptional regulation of LOXL2 as one mechanism responsible for LOXL2 secretion in activated HSC.

**Fig. 4:**
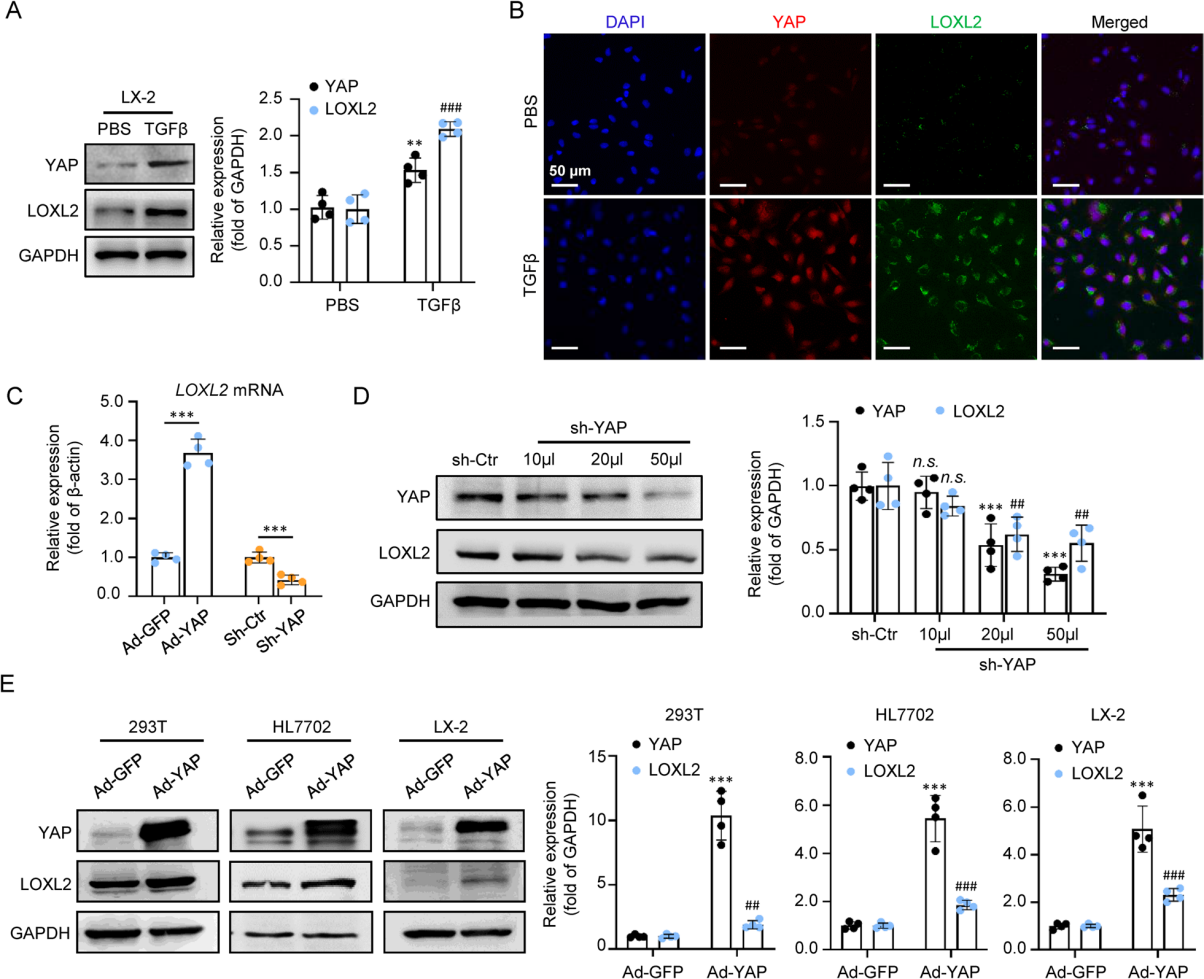
IdentifyingLOXL2 as a downstream target gene of YAP in LX-2. **A** Western blot analysis and quantification of YAP and LOXL2 protein expression in PBS and TGFβ activated LX-2 cells (n = 4; **p < .01, ^###^p < .001). **B** Immunofluorescence staining images of YAP (Red) and LOXL2 (Green) protein in PBS and TGFβ activated LX-2 cells. Scale bars, 50 μm. **C** qRT-PCR analysis for LOXL2 mRNA in Ad-GFP, Ad-YAP, sh-Ctr, or transfected LX-2 cells (n = 4; ***p < .001). **D** Western blot analysis and quantification of YAP and LOXL2 protein expression in sh-Ctr or sh-YAP transfected LX-2 cells (n = 4; ***p < .001, ^##^p < .01, and n.s., Not significant). **E** Western blot analysis and quantification of YAP and LOXL2 protein expression in Ad-GFP or Ad-YAP transfected 293T, HL7702, and LX-2 cells (n = 4; ***p < .001, and ^##^p < .01, and ^###^p < .001)

TGFβ is known to induce the activation of HSCs [23]. We further examined the expression of YAP and LOX in TGFβ activated LX-2 cells. As shown in Fig. 4A, TGFβ treatment induced YAP and LOX expression in LX-2. Immunofluorescence double staining showed a positive correlation between YAP and LOXL2 in TGFβ activated HSC (Fig. 4B). qRT-PCR showed that LOXL2 mRNA level in LX-2 cells was significantly increased by Ad-YAP and decreased by sh-YAP (Fig. 4C). Knockdown of YAP expression by shRNA dose-dependently downregulated LOXL2 expression in LX-2 (Fig. 4D). To provide causal evidence of the impact of YAP on LOXL2 expression regulation, Ad-YAP was used to transfect 293T, HL7702, and LX-2 cells, respectively, and found that induced YAP overexpression increased LOXL2 protein expression (Fig. 4E). These results suggest a close relationship between YAP overexpression and LOXL2 upregulation, and LOXL2 was identified as an essential downstream target gene of YAP in HSC.

### MSC-ex downregulated YAP/LOXL2 expression through exosomal miR-27b-3p

**Fig. 5:**
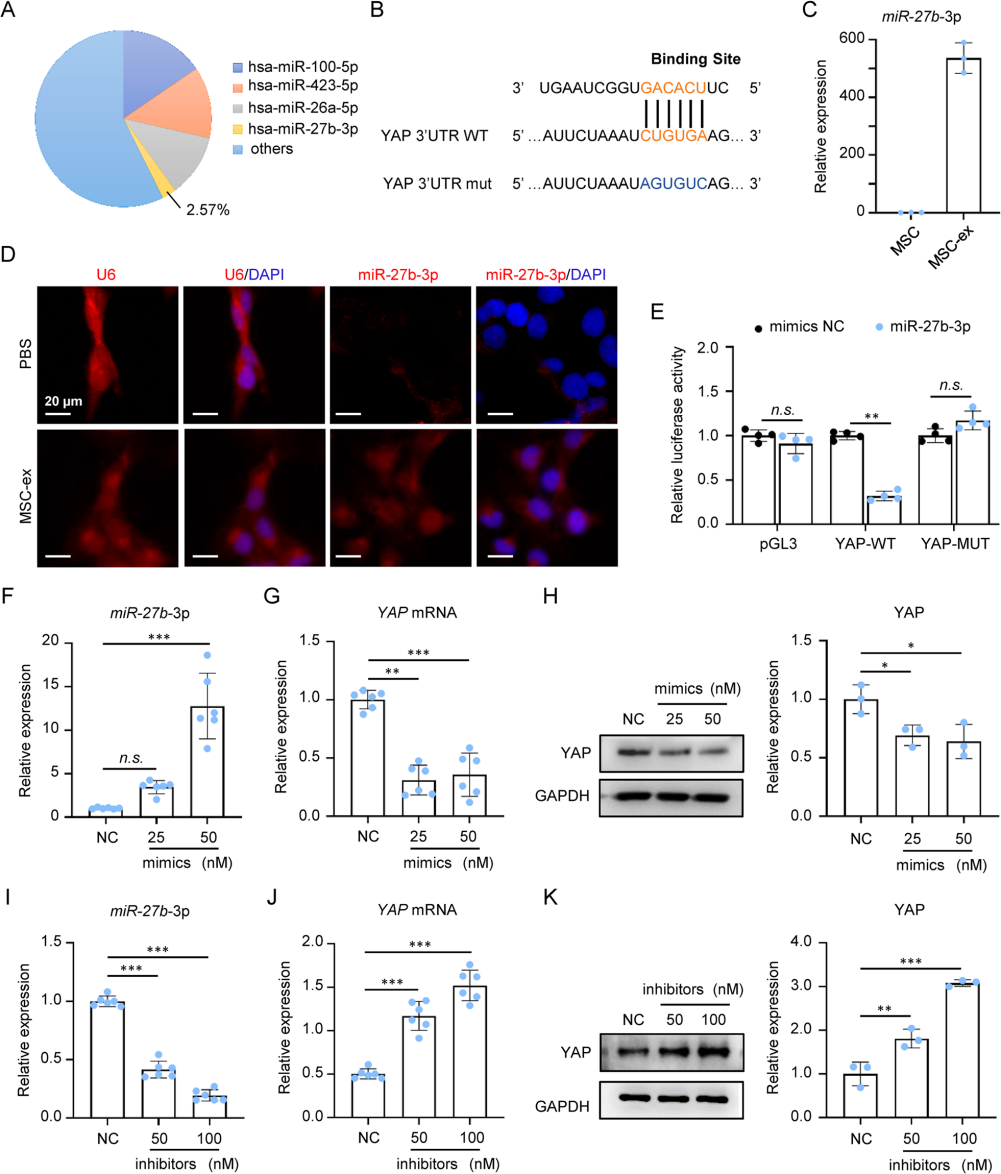
miR-27b-3p enriched in MSC-ex and downregulated YAP expression. **A** Relative percentage of miRNAs in total miRNA reads. **B** The predicted binding site of miR-27b-3p targeting the 3ʹ-UTR of YAP. **C** qRT-PCR of miR-27b-3p in isolated MSC and MSC-ex (n = 3). **D** FISH analysis of U6 and miR-27b-3p in PBS or MSC-ex treated LX-2 cells. Scale bars, 20 µm. **E** Luciferase reporter assay showed YAP as a target of miR-27b-3p (n = 4; **p < .01, n.s., Not significant). NC, negative control; WT, wide type; MUT, mutant. **F** qRT-PCR of miR-27b-3p in miR-27b-3p mimics transfected LX-2 (n = 6; ***p < .001, n.s., Not significant). **G** qRT-PCR of YAP mRNA in miR-27b-3p mimics transfected LX-2 (n = 6; **p < .01, ***p < .001). **H** Western blot and quantification of YAP protein in miR-27b-3p mimic transfected LX-2 (n = 3; *p < .05). **I** qRT-PCR of miR-27b-3p in miR-27b-3p inhibitors transfected LX-2 (n = 6; ***p < .001). **J** qRT-PCR of YAP mRNA in miR-27b-3p inhibitors transfected LX-2 (n = 6; ***p < .001). **K** Western blot and quantification of YAP protein in miR-27b-3p inhibitors transfected LX-2 (n = 3; **p < .01, ***p < .001)

YAP is a key transcription co-factor in the Hippo pathway, and its dysfunction is involved in the pathogenesis of various diseases, including tissue fibrosis [24]. To elucidate the active molecules by which MSC-ex inhibited LOXL2 expression and suppressed hepatic fibrosis progression, the miRNA expression profile of MSC-ex was screened by miRNA-seq (Oebiotech, OE2015H1459). We found miR-100-5p, miR-423-5p, miR-26a-5p, and miR-27b-3p, accounted for approximately 43% of the total miRNA reads (Fig. 5A; source: Key Laboratory of Medical Science and Laboratory Medicine of Jiangsu Province, School of Medicine, Jiangsu University). The bioinformatics database TargetScan was applied to explore the miRNAs that target YAP, and we found that 3ʹ UTR of YAP is predicted to be a binding-target sequence of miR-27b-3p (Fig. 5B). qRT-PCR suggested that miR-27b-3p was enriched in MSC-ex (Fig. 5C). ddPCR showed that the number of miR-27b-3p in MSC-ex is 742 copies/10^4^ particles (Additional file 4: Figure S4). miR-27b-3p was also detected in MSC-ex treated LX-2 cells by FISH, which showed that exosomal miR-27b-3p could be delivered to LX-2 cells (Fig. 5D). Furthermore, to assess whether there was a direct interaction between miR-27b-3p and YAP, luciferase reporter plasmid containing either wild-type or mutant 3ʹ UTRs of YAP was constructed. Luciferase reporter assay showed that transfection of miR-27b-3p mimics could significantly reduce the luciferase activity of wide-type YAP 3ʹ UTRs compared with the mutant 3ʹ UTRs construct (Fig. 5E). To further clarify whether the reduction of YAP was regulated by miR-27b-3p, YAP expression was investigated in LX-2 transfected with miR-27b-3p mimics or miR-27b-3p inhibitor. qRT-PCR analysis demonstrated that 25 nM and 50 mM mimics showed a 3.6- and 11.4-fold increase in miR-27b-3p expression (Fig. 5F). miR-27b-3p overexpression reduced YAP mRNA and protein expression dose-dependently (Fig. 5G, H). Consistently, 25 nM and 50 nM inhibitors decreased miR-27b-3p expression, and miR-27b-3p knockdown increased YAP mRNA and protein expression in LX-2 (Fig. 5I–K). These results suggested that MSC-ex exosomal miR-27b-3p can transfer into LX-2, while miR-27b-3p played an important role in YAP down-regulation.

**Fig. 6:**
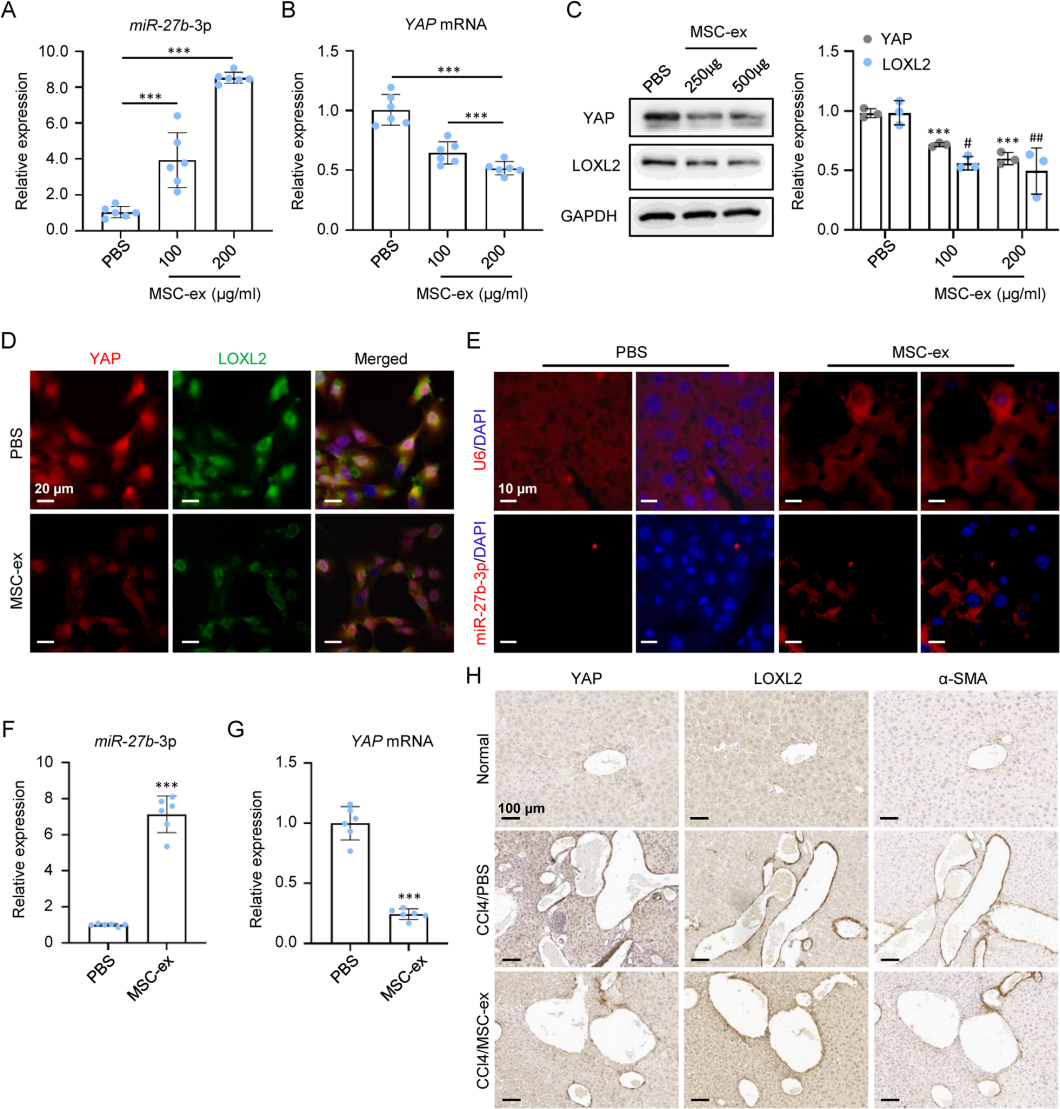
MSC-ex increased miR-27b-3p expression and downregulated YAP/LOXL2 expression. **A** qRT-PCR analysis of miR-27b-3p in PBS or MSC-ex (100 or 200 μg/mL) treated LX-2 cells (n = 6; ***p < .001). **B** qRT-PCR analysis of YAP mRNA in PBS or MSC-ex (100 or 200 μg/mL) treated LX-2 cells (n = 6; ***p < .001). **C** Western blot analysis and quantification of YAP protein in PBS or MSC-ex (100 or 200 μg/mL) treated LX-2 cells (n = 3; ***p < .001, ^#^p < .05, and ^##^p < .01). **D** Immunofluorescence staining images of YAP (Red) and LOXL2 (Green) protein in PBS or MSC-ex treated LX-2 cells. Scale bars, 20 μm. **E** FISH analysis of U6 and miR-27b-3p in PBS or MSC-ex treated fibrotic livers. Scale bars, 10 µm. **F** qRT-PCR analysis of miR-27b-3p in PBS or MSC-ex treated fibrotic livers (n = 6; **p < .01). **G** qRT-PCR analysis of YAP mRNA in PBS or MSC-ex treated fibrotic livers (n = 6; ***p < .001). **H** Immunohistochemistry staining for YAP, LOXL2, and α-SMA in PBS or MSC-ex treated fibrotic livers. Scale bars, 50 μm

To determine whether exosomal miR-27b-3p can reduce YAP upregulated LOXL2 expression, we examined YAP and LOXL2 expression in activated LX-2 cells and fibrotic livers before and after MSC-ex treatment. In LX-2 cells, qRT-PCR showed that treatment of MSC-ex significantly increased the expression of miR-27b-3p and decreased YAP mRNA expression dose-dependently (Fig. 6A, B). As shown in Fig. 6C, the western blot confirmed that MSC-ex treatment decreased YAP and LOXL2 protein expression in LX-2 cells. Double immunofluorescence also revealed that nuclear YAP localization and LOXL2 expression were aberrantly reduced in MSC-ex-treated LX-2 cells (Fig. 6D). Consistent with in vitro data, the FISH assay indicated that miR-27b-3p was detected in MSC-ex-treated fibrotic livers (Fig. 6E). MSC-ex significantly increased the expression of miR-27b-3p and decreased YAP mRNA expression (Fig. 6F, G). Immunohistochemistry showed that MSC-ex could reduce YAP, LOXL2, and activated HSC marker α-SMA expression in the portoportal regions of the fibrotic livers (Fig. 6H). Thus, these data indicated that MSC-ex might suppress YAP/LOXL2 expression through exosomal miR-27b-3p transfer in HSC cells.

### miR-27b-3p knockdown mitigated the YAP/LOXL2 inhibition efficacy of MSC-ex

**Fig. 7:**
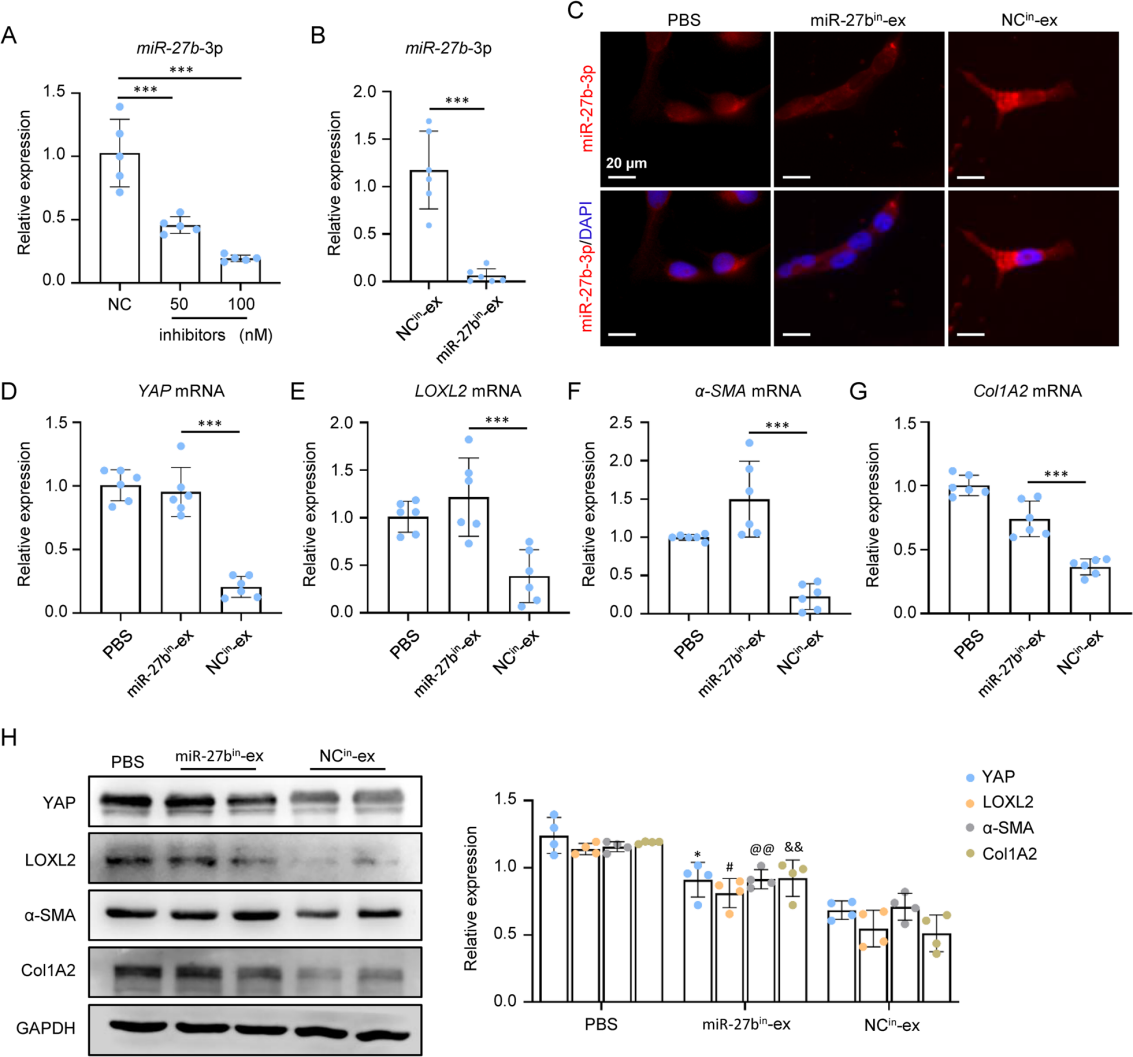
miR-27b-3p knockdown mitigated YAP/LOXL2 inhibition efficacy of MSC-ex. **A** qRT-PCR analysis of miR-27b-3p in negative control inhibitors (NC) or miR-27b-3p inhibitors transfected MSC (n = 6; ***p < .001). **B** qRT-PCR analysis of miR-27b-3p in exosomes isolated from NC inhibitors transfected MSC (NC^in^-ex), or miR-27b-3p inhibitors transfected MSC (miR-27b^in^-ex) (n = 6; ***p < .001). C. FISH analysis of miR-27b-3p in PBS, 200 μg/mL NC^in^-ex or miR-27b^in^-ex treated LX-2 cells. Scale bars, 20 µm. **D**–**G** qRT-PCR analysis of YAP, LOXL2, α-SMA and Col1A2 mRNA in PBS, 200 μg/mL NC^in^-ex or miR-27b^in^-ex treated LX-2 cells (n = 6; ***p < .001). **H** Western blot analysis and quantification of YAP, LOXL2, α-SMA and Col1A2 protein in PBS, NC^in^-ex or miR-27b^in^-ex treated LX-2 cells (n = 4; *p < .05, ^#^p < .05, ^@@^p < .01, and ^&&^p < .01)

To further determine whether miR-27b-3p was involved in MSC-ex-mediated YAP and LOXL2 inhibition, MSC were transfected with miR-27b-3p inhibitors to construct miR-27b-3p inhibitor transfected MSC (Fig. 7A). As shown in Fig. 7B, miR-27b-3p expression was decreased in exosomes isolated from miR-27b-3p inhibitor transfected MSC (miR-27b^in^-ex) compared with the exosomes isolated from negative control (NC) miRNA transfected MSC (NC^in^-ex). When NC^in^-ex or miR-27b^in^-ex were incubated with LX-2 cells, FISH showed that the knockdown of miR-27b-3p markedly reversed MSC-ex-induced up-regulation of miR-27b-3p (Fig. 7C). Furthermore, we examined the YAP, LOXL2, α-SMA, and Col1A2 levels in NC-ex or miR-27b^in^-ex treated LX-2 cells. As shown in Fig. 7D–G, miR-27b^in^-ex markedly reversed the decrease of YAP, LOXL2, α-SMA, and Col1A2 mRNA in the NC^in^-ex treatment group. NC^in^-ex mediated down-regulation of YAP, LOXL2, α-SMA, and Col1A2 protein was also reversed by miR-27b^in^-ex (Fig. 7H). These results indicated that MSC-ex downregulated YAP/LOXL2 expression via miR-27b-3p.

**Fig. 8:**
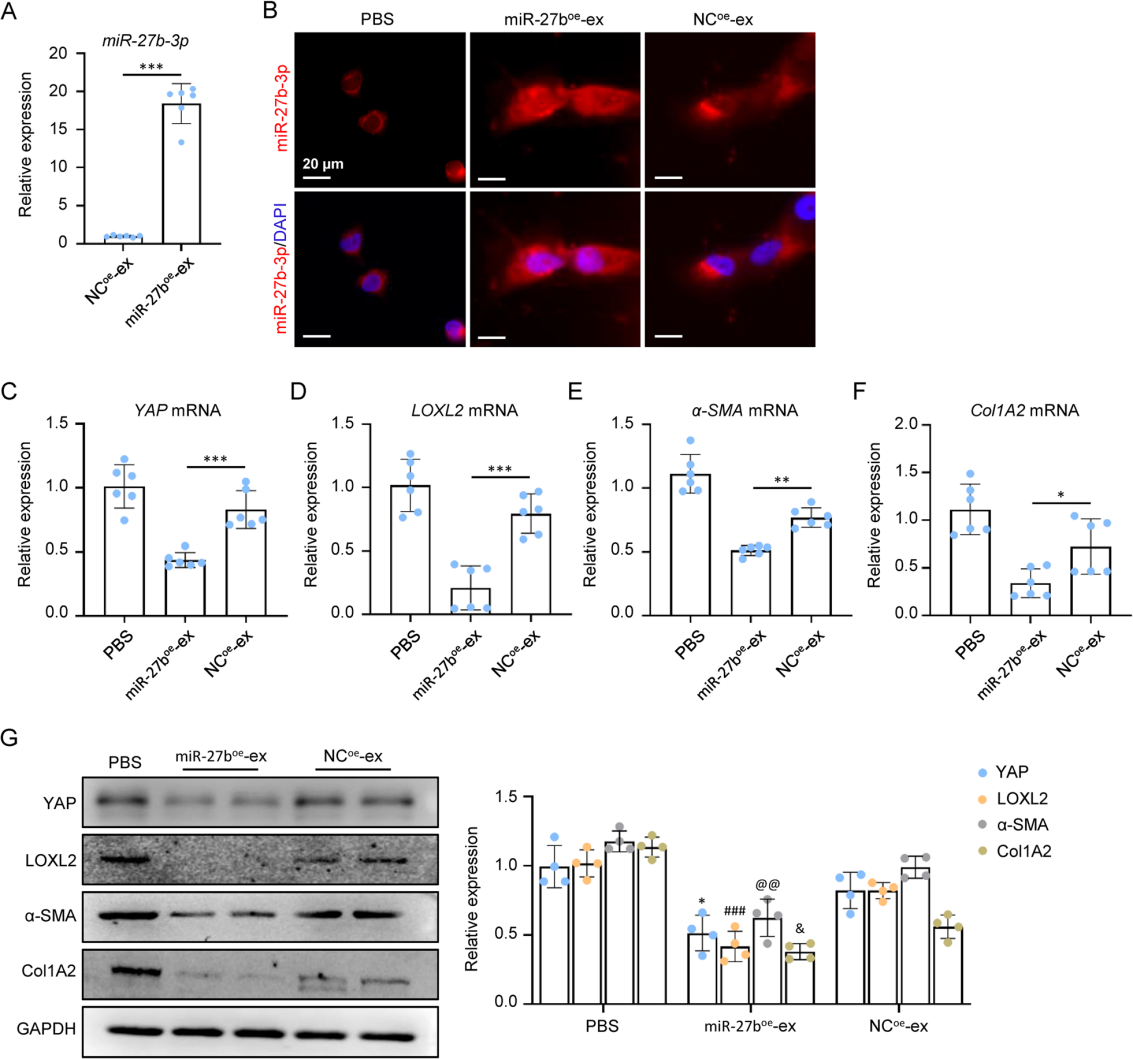
miR-27b-3p overexpression enhanced YAP/LOXL2 inhibition efficacy of MSC-ex. **A** qRT-PCR of miR-27b-3p in exosomes isolated from negative control (NC) mimics overexpressed MSC-ex (NC^oe^-ex) or miR-27b-3p mimics overexpressed MSC-ex (miR-27b^oe^-ex) (n = 6; ***p < .001). **B** FISH of miR-27b-3p in PBS, 50 μg/mL NC^oe^-ex or miR-27b^oe^-ex treated LX-2. Scale bars, 20 µm. **C**–**F** qRT-PCR of YAP, LOXL2, α-SMA and Col1A2 mRNA in PBS, 50 μg/mL NC^oe^-ex or miR-27b^oe^-ex treated LX-2 (n = 6; *p < .05, **p < .01, and ***p < .001). **G** Western blot analysis and quantification of YAP, LOXL2, α-SMA and Col1A2 protein in PBS, NC^oe^-ex or miR-27b^oe^-ex treated LX-2 (n = 4; *p < .05, ^###^p < .001, ^@@^p < .01, and ^&^p < .05)

To further identify the effect of MSC-ex-derived miR-27b-3p on YAP/LOXL2 downregulation, miR-27b-3p was passively loaded into the MSC-ex by sonication method. qRT-PCR suggested that miR-27b-3p was significantly increased in miR-27b-3p overexpressed MSC-ex (miR-27b^oe^-ex) (Fig. 8A). In miR-27b^oe^-ex treated LX-2 cells, miR-27b-3p was significantly increased compared with the negative control miRNA overexpressed MSC-ex (NC^oe^-ex) (Fig. 8B). NC-ex downregulated YAP, LOXL2, α-SMA, and Col1A2 mRNA expression was consistently enhanced by miR-27b^oe^-ex (Fig. 8C–F). Western blot also showed that miR-27b^oe^-ex markedly increased the inhibition of YAP, LOXL2, α-SMA, and Col1A2 protein expression by NC^oe^-ex (Fig. 8G). These results indicated that miR-27b-3p modification could enhance the inhibitory effect of MSC-ex on YAP/LOXL2 expression.

## Discussion

Liver fibrosis is a continuous wound-healing process of chronic injury. It results from excessive deposition and degreased degradation of the extracellular matrix (ECM) [2]. LOXL2 is crucial in collagen crosslinking, ECM remodeling, myofibroblasts activation, and rendering [8]. In this content, anti-LOXL2 represents an attractive antifibrotic strategy for liver fibrosis. In the present study, we described a previously unknown role for MSC-ex in LOXL2 inhibition in the HSC of the CCl4-induced liver fibrosis mouse model. Mechanistically, miR-27b-3p is abundant in MSC-ex. MSC-ex reduced YAP-activated LOXL2 expression by releasing miR-27b-3p, inhibiting liver fibrosis, which indicates that MSC-ex may represent a new strategy for treating liver fibrosis.

Recently, inhibition of LOXL2 by small-molecule inhibitors and monoclonal antibodies has decreased fibrosis and increased survival in rodent models of liver fibrosis [8, 25]. However, a humanized anti-LOXL2 antibody Simtuzumab did not demonstrate a clinical benefit in patients with primary sclerosing cholangitis and NASH [12–14]. The reasons for the Simtuzumab failure include a weak LOXL2 antagonist effect in vivo. It is also unclear whether simtuzumab can penetrate liver scar and increase intracellular drug activity [15]. In this study, our data showed that most of the DiR labeled MSC-ex targeted the injured liver and were located in the portoportal regions of the fibrotic liver. MSC-ex may be a cell-permeable drug targeting LOXL2 in vivo. BAPN has been extensively used as a non-selective LOX inhibitor in multiple fibrosis models [7, 26, 27]. We next analyzed the inhibition of LOXL2 activity and collagen accumulation of MSC-ex and *β*-aminopropionitrile (BAPN). In comparison with BAPN treatment, MSC-ex achieved better anti-LOXL2 results in vivo. Accordingly, MSC-ex (25 mg/kg) and BAPN treatment reduced collagen deposition, respectively. Our results provide, for the first time, evidence that MSC-ex could serve as a novel agent for anti-LOXL2 inhibition in liver fibrosis.

MSC can be obtained from bone marrow and other fetal or postnatal tissues, including adipose tissue, umbilical cord blood, and the Wharton’s jelly of the umbilical cord [28]. Among these sources, the umbilical cord is an attractive source of MSC because of its abundant tissue source, less immunogenic, and less invasive than other sources [29, 30]. Furthermore, exosomes from umbilical cord-derived MSC were more effective than adipose-derived MSC or bone marrow-derived MSC in repairing tissue damage [31]. Therefore, we chose the human umbilical cord for this study. To investigate the role of MSC-ex in LOXL2 regulation, we injected MSC-ex directly into the liver using a mouse model of CCl4-mediated liver fibrosis. Although species differences do not allow us to conclude the therapeutic potential of human liver injury, studies in mice suggest that MSC-ex may be an effective strategy for reducing collagen deposition.

Activated HSC and myofibroblasts are primary cellular sources of LOXL2 in liver fibrogenesis [9]. Our previous studies have shown that MSC-ex can inhibit the activation of HSC and collagen deposition, but the mechanism of action is still unclear [32]. In this study, we found that MSC-ex downregulated LOXL2 and α-SMA expression. These results indicated that HSC may serve as the cellular target of MSC-ex. Thus, HSC was chosen for further analysis of MSC-ex-mediated anti-LOXL2 regulation. Tremendous effort has been put into defining the biological functions of LOXL2 in remodeling extracellular matrix (ECM) and the cross-linking of collagen in liver fibrosis [9, 15]. However, few studies examined the molecular pathways involved in LOXL2 regulations. Several mechanisms of LOXL2 regulation have been proposed in cancer development and cardiac fibrosis. Under the stress of inflammation, NF-κB induced LOXL2 mRNA expression, triggering fibroblast activation [33]. TGFβ may act via SMAD to regulate LOX expression, increasing cardiac fibrosis [34]. Our study showed that MSC-ex could inhibit TGFβ induced YAP and LOXL2 expression in TGFβ activated HSC.

For the first time, we identified LOXL2 as a direct downstream target gene of YAP, which positively regulated LOXL2 transcription in HSC. YAP is a well-defined downstream effector in the Hippo pathway that promotes HSC activation when translocated to the nucleus during liver fibrosis [24]. YAP activation increased the stiffness of ECM, which further stimulated the activation of YAP in turn, forming a feed-forward loop to promote liver fibrosis [24]. However, the mechanism YAP regulates LOXL2 in fibrotic tissues and hepatic stellate cells has not been described. We showed that YAP promotes LOXL2 transcriptional regulation by binding to the LOXL2 promoter sequence in LX-2, L-02, and 293T cells. In serial sections of hepatic fibrosis, we also found that the expressions of YAP and LOXL2 were pathologically correlated before and after MSC-ex action. These results indicated that YAP may be a potential target of LOXL2 inhibition or anti-fibrosis therapy. MSC-ex may suppress hepatic fibrosis progression by downregulating YAP transactivated LOXL2 expression.

Exosomes can specifically target recipient cells to exchange miRNAs, mRNAs, and proteins to trigger downstream signaling events [19]. miRNAs are considered one of the main MSC-ex cargoes from various sources. Many studies have reported the therapeutic efficacy of MSC-ex with well-characterized miRNAs in liver disease. miR-182-5p from hypoxia preconditioned MSC-derived exosome facilitates macrophage polarization during liver regeneration by modulating the FOXO1/TLR4 signaling pathway [35]. miR-148a released by MSC-ex regulates the function of intrahepatic macrophages and prevents liver fibrosis through the KLF6/STAT3 signaling pathway [36]. Since targeting YAP can suppress HSC activation and LOXL2 expression, we searched the miRNAs that targeted YAP with the bioinformatic database TargetScan and analyzed miRNAs enriched in MSC-ex. Our results showed that MSC-ex-derived exosomal miR-27b-3p could downregulate YAP/LOXL2 signaling and exert an anti-HSC activation effect in HSC. miR-27b-3p knockdown mitigated the YAP/LOXL2 inhibition efficacy of MSC-ex. miR-27b-3p overexpression enhanced the inhibitory effect of MSC-ex on YAP/LOXL2 expression. Thus, we concluded that miR-27b-3p plays a key role in YAP/LOXL2 of HSC.

While this study offers valuable insights, it is essential to acknowledge its limitations. One such challenge is the need for more knowledge regarding other active proteins or miRNAs in MSC-ex that may inhibit HSC activation and liver fibrosis. Furthermore, it remains to be seen which target molecules MSC-ex acts on besides LOXL2. Additionally, our study found that after in vivo injection, MSC-ex was localized in HSC and taken up by hepatocytes, endothelial cells, and macrophages. MSC-ex may carry other proteins or miRNAs, which may regulate these important target cells in the liver and inhibit liver fibrosis. Further research into these questions will significantly improve our understanding of the mechanisms behind the anti-fibrotic effects of MSC-ex.

**Fig. 9:**
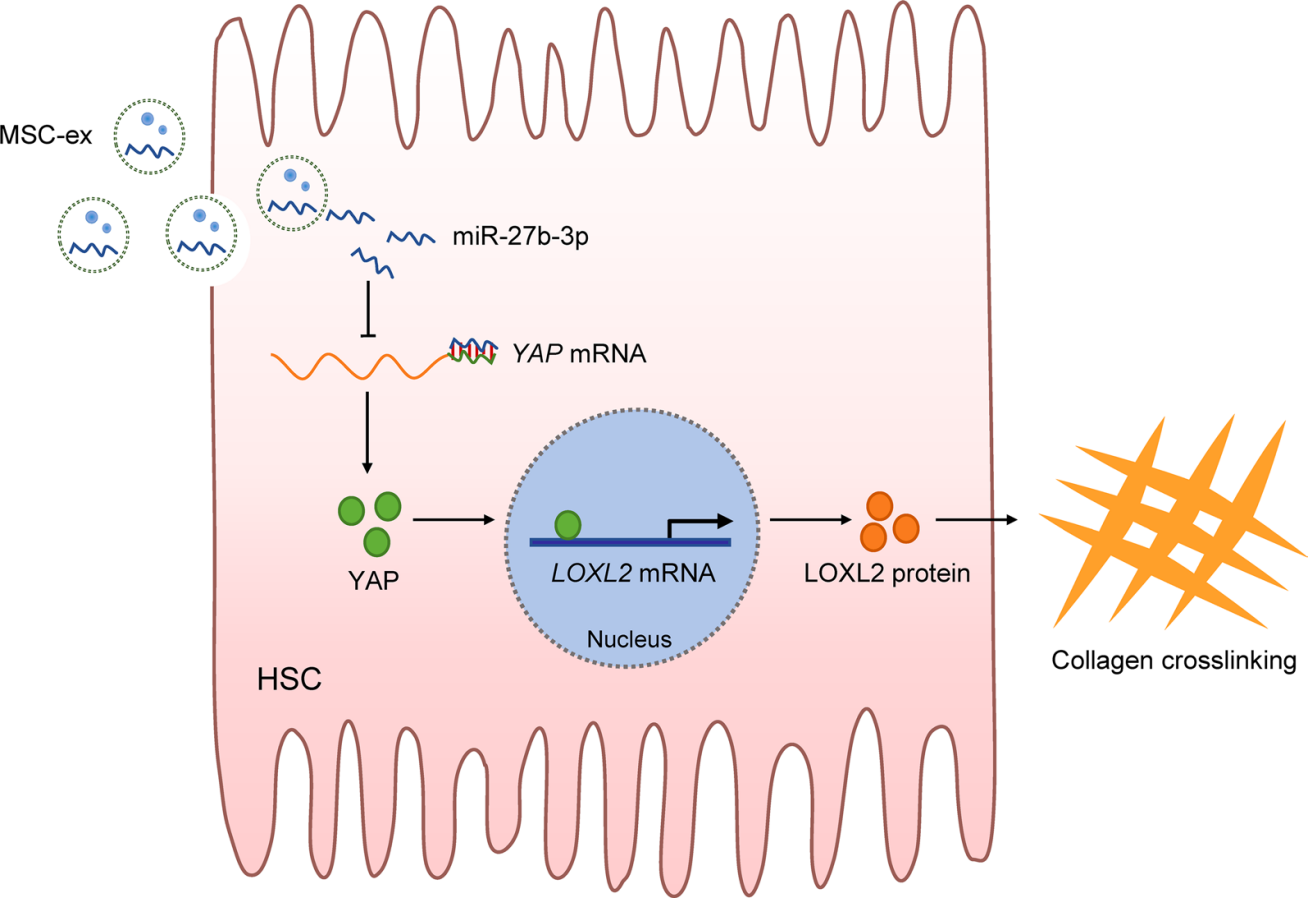
Schematic representation of the mechanism of miR-27b-3p enriched MSC-ex for the downregulation of LOXL2. MSC-ex can deliver miR-27b-3p into HSCs and suppress YAP mRNA expression. Reduced YAP decreases LOX2 transcription and protein expression, reducing collagen crosslinking. MSC-ex, Mesenchymal stem cell-derived exosome. HSCs, hepatic stellate cells

In summary, we reported that injected MSC-ex can locate in the HSC of fibrotic liver and substantially reduce LOXL2 expression, collagen matrix deposition, fibrosis progression, and direct HSC activation. MSC-ex-derived exosomal miR-27b-3p may target YAP, reducing YAP transcriptional-activated LOXL2 expression and inhibiting collagen crosslinking in vitro and in vivo (Fig. 9).

## Supplementary Information

**Additional file 1: Figure S1.** Quantified fluorescence intensity of mice liver from PBS or DiR labeled MSC-ex group.

**Additional file 2: Figure S2.** Safety evaluation of MSC-ex in health mice. A. Haematoxylin and eosin (HE) staining of cardiac, liver, spleen, lung, and kidney tissues in healthy mice intravenously injected with PBS or MSC-ex. Scale bars, 40 µm. B. The levels of liver function indicators (ALT, alanine aminotransferase; AST, aspartate aminotransferase), cardiac function indicators (LDH, lactate dehydrogenase), and renal function indicators UREA in sera from PBS or MSC-ex treated mice.

**Additional file 3: Figure S3.** Uptake of PKH26-MSC-ex in F4/80^+^ Kupffer cells, albumin^+^ hepatocytes, or CD31^+^ endothelial cells in fibrotic mouse liver. A. PKH26-labeled MSC-ex was taken up by F4/80^+^ Kupffer cells in fibrotic mouse liver 24 h post intravenous injection. Scale bars, 100 µm. B. PKH26-labeled MSC-ex was taken up by CD31^+^ endothelial cells in fibrotic mouse liver. Scale bars, 100 µm. C. PKH26-labeled MSC-ex was taken up by albumin^+^ hepatocytes in fibrotic mouse liver. Scale bar, 100 µm.

**Additional file 4: Figure S4.** Number of miR27b-3p in MSC-ex detected by ddPCR. A. Fluorescence amplitude of miR-27b-3p in MSC-ex using ddPCR. 125 ng RNA from MSC-ex (10^4^ particles) was used to generate cDNA by reverse transcription. ddPCR was performed using cDNA from 125 ng RNA in a total PCR system of 25 μL. The average concentration of miR-27b-3p is represented in copies/μL. B. ddPCR determined miR-27b-3p copies of MSC-ex (104 particles). Four parallel samples were tested.

## Data Availability

All data in this study are available from the corresponding author upon reasonable request.

## Abbreviations

MSC-ex: Mesenchymal stem cells derived exosomes
HSC: Hepatic stellate cells
MSC: Mesenchymal stem cells
TGFβ: Transforming growth factor-β
LOXL2: Lysyl oxidase-like 2
YAP: Yes-associated protein
α-SMA: α-Smooth muscle actin
BAPN: β-Aminopropionitrile

## References

[1] Shah PA, Patil R, Harrison SA (2023). NAFLD-related hepatocellular carcinoma: the growing challenge. Hepatology.

[2] Asrani SK (2019). Burden of liver diseases in the world. J Hepatol.

[3] Zanetto A (2023). Hemostasis in cirrhosis: understanding destabilising factors during acute decompensation. J Hepatol.

[4] Dongiovanni P (2017). Insulin resistance promotes Lysyl Oxidase Like 2 induction and fibrosis accumulation in non-alcoholic fatty liver disease. Clin Sci (Lond).

[5] Pollheimer MJ (2018). Lysyl oxidase-like protein 2 (LOXL2) modulates barrier function in cholangiocytes in cholestasis. J Hepatol.

[6] Gharib AM (2017). Magnetic resonance elastography shear wave velocity correlates with liver fibrosis and hepatic venous pressure gradient in adults with advanced liver disease. Biomed Res Int.

[7] Hutchinson JH (2017). Small molecule lysyl oxidase-like 2 (LOXL2) inhibitors: the identification of an inhibitor selective for LOXL2 over LOX. ACS Med Chem Lett.

[8] Barry-Hamilton V (2010). Allosteric inhibition of lysyl oxidase-like-2 impedes the development of a pathologic microenvironment. Nat Med.

[9] Magdaleno F, Trebicka J (2017). Selective LOXL2 inhibition: potent antifibrotic effects in ongoing fibrosis and fibrosis regression. Gut.

[10] Ikenaga N (2017). Selective targeting of lysyl oxidase-like 2 (LOXL2) suppresses hepatic fibrosis progression and accelerates its reversal. Gut.

[11] Raghu G (2017). Efficacy of simtuzumab versus placebo in patients with idiopathic pulmonary fibrosis: a randomised, double-blind, controlled, phase 2 trial. Lancet Respir Med.

[12] Muir AJ (2019). Simtuzumab for primary sclerosing cholangitis: phase 2 study results with insights on the Natural History of the Disease. Hepatology.

[13] Harrison SA (2018). simtuzumab is ineffective for patients with bridging fibrosis or compensated cirrhosis caused by nonalcoholic steatohepatitis. Gastroenterology.

[14] Meissner EG (2016). Simtuzumab treatment of advanced liver fibrosis in HIV and HCV-infected adults: results of a 6-month open-label safety trial. Liver Int.

[15] Chen W (2020). Lysyl oxidase (LOX) family members: rationale and their potential as therapeutic targets for liver fibrosis. Hepatology.

[16] Gao Y, Yin X, Ren X (2022). Advance of mesenchymal stem cells in chronic end-stage liver disease control. Stem Cells Int.

[17] Xu X (2010). Isolation of cancer stem cells from transformed human mesenchymal stem cell line F6. J Mol Med (Berl).

[18] Liang W (2021). Mesenchymal stem cells as a double-edged sword in tumor growth: focusing on MSC-derived cytokines. Cell Mol Biol Lett.

[19] Psaraki A (2022). Extracellular vesicles derived from mesenchymal stem/stromal cells: the regenerative impact in liver diseases. Hepatology.

[20] Lai RC, Chen TS, Lim SK (2011). Mesenchymal stem cell exosome: a novel stem cell-based therapy for cardiovascular disease. Regen Med.

[21] Yan Y (2017). hucMSC exosome-derived GPX1 Is required for the recovery of hepatic oxidant injury. Mol Ther.

[22] Jiang W (2018). Human umbilical cord MSC-derived exosomes suppress the development of CCl(4)-induced liver injury through antioxidant effect. Stem Cells Int.

[23] Dewidar B (2019). TGF-beta in hepatic stellate cell activation and liver fibrogenesis-updated 2019. Cells.

[24] Mitten EK, Baffy G (2022). Mechanotransduction in the pathogenesis of non-alcoholic fatty liver disease. J Hepatol.

[25] Findlay A (2021). An activity-based bioprobe differentiates a novel small molecule inhibitor from a LOXL2 antibody and provides renewed promise for anti-fibrotic therapeutic strategies. Clin Transl Med.

[26] Liu SB (2016). Lysyl oxidase activity contributes to collagen stabilization during liver fibrosis progression and limits spontaneous fibrosis reversal in mice. FASEB J.

[27] Iwasaki A (2016). Molecular mechanism responsible for fibronectin-controlled alterations in matrix stiffness in advanced chronic liver fibrogenesis. J Biol Chem.

[28] Todtenhaupt P (2022). Mesenchymal stromal cells as a tool to unravel the developmental origins of disease. Trends Endocrinol Metab.

[29] Rodriguez-Eguren A (2022). Human umbilical cord-based therapeutics: stem cells and blood derivatives for female reproductive medicine. Int J Mol Sci.

[30] Zhang W (2022). Comparison of therapeutic effects of mesenchymal stem cells from umbilical cord and bone marrow in the treatment of type 1 diabetes. Stem Cell Res Ther.

[31] Wang ZG (2020). Comprehensive proteomic analysis of exosomes derived from human bone marrow, adipose tissue, and umbilical cord mesenchymal stem cells. Stem Cell Res Ther.

[32] Li T (2013). Exosomes derived from human umbilical cord mesenchymal stem cells alleviate liver fibrosis. Stem Cells Dev.

[33] Tashkandi MM (2020). LOXL2 promotes aggrecan and gender-specific anabolic differences to TMJ cartilage. Sci Rep.

[34] Lu M (2019). Induction of LOX by TGF-beta1/Smad/AP-1 signaling aggravates rat myocardial fibrosis and heart failure. IUBMB Life.

[35] Xu J (2022). Hypoxic bone marrow mesenchymal stromal cells-derived exosomal miR-182-5p promotes liver regeneration via FOXO1-mediated macrophage polarization. FASEB J.

[36] Tian S (2022). Mesenchymal stem cell-derived exosomes protect against liver fibrosis via delivering miR-148a to target KLF6/STAT3 pathway in macrophages. Stem Cell Res Ther.

