# Supplementary figures and images for: Mesenchymal stem cell-derived exosomal miR-27b-3p alleviates liver fibrosis via downregulating YAP/LOXL2 pathway
Source: J Nanobiotechnology. 2023 Jun 16;21:195. doi: 10.1186/s12951-023-01942-y (PMC10273609; doi:10.1186/s12951-023-01942-y)

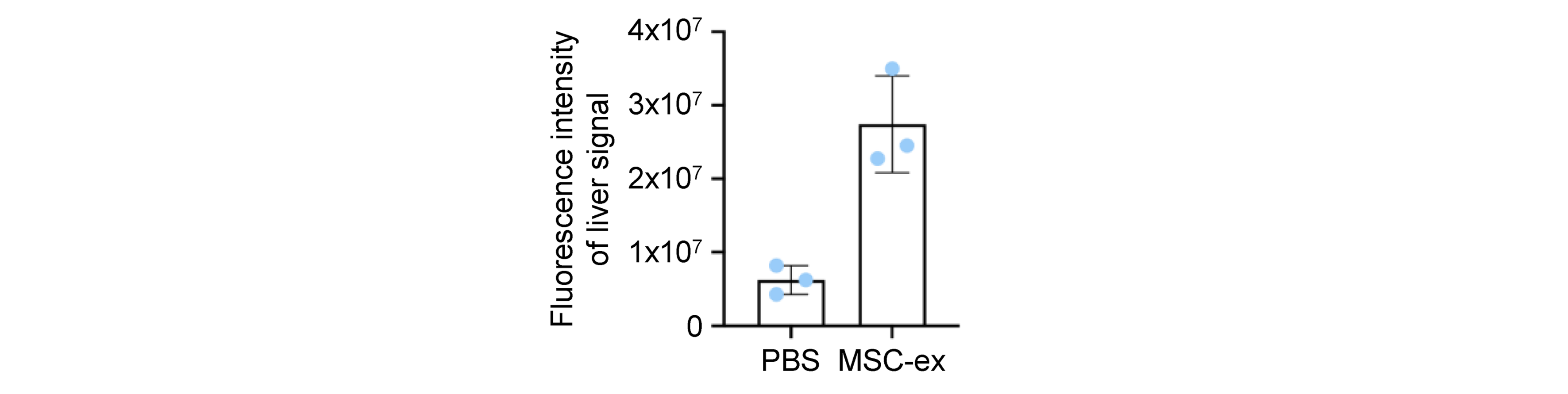

Supplement: Supplementary file 1 — Additional file 1: Figure S1. Quantified fluorescence intensity of mice liver from PBS or DiR labeled MSC-ex group. [file 12951_2023_1942_MOESM1_ESM.tiff]

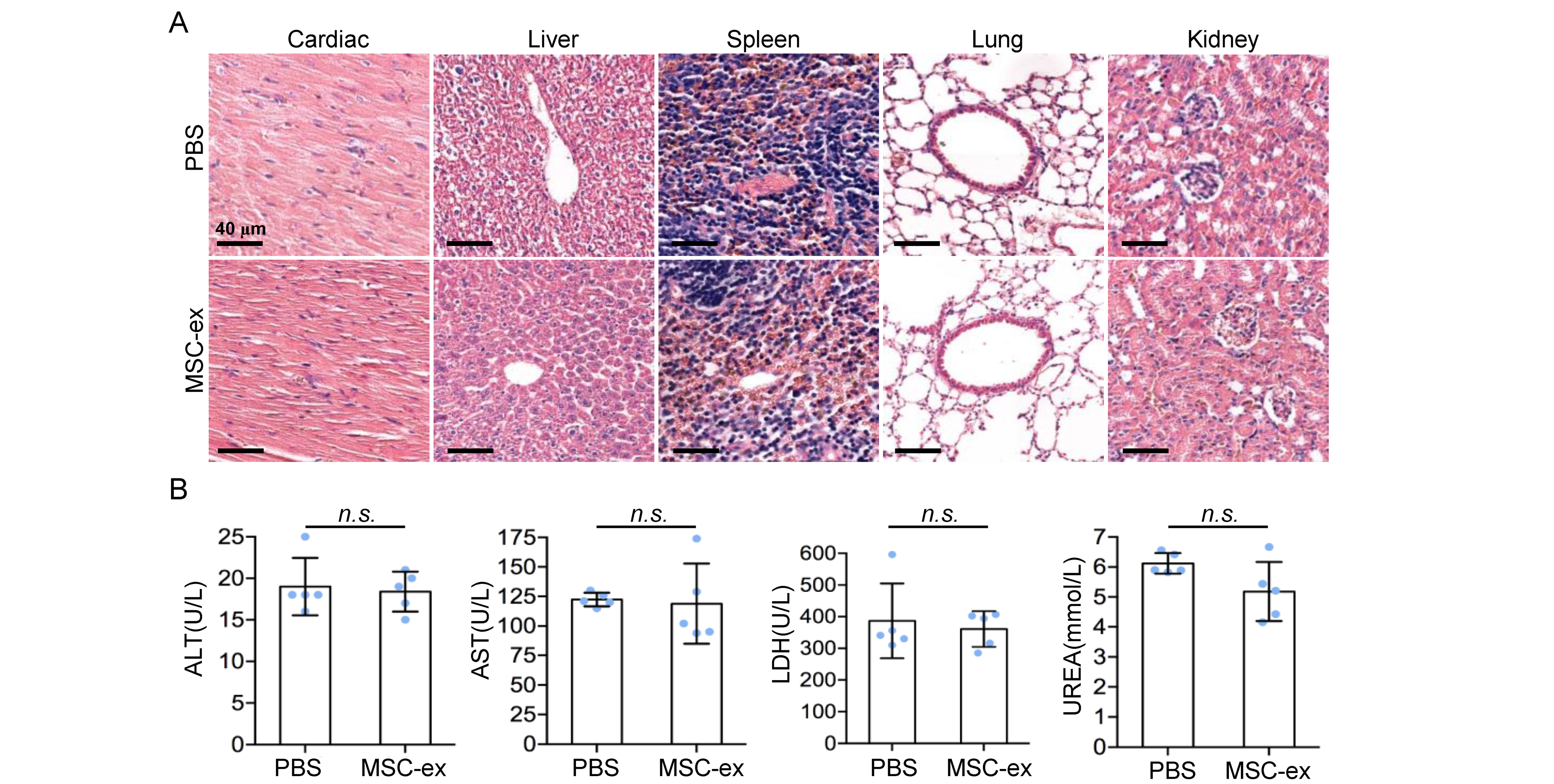

Supplement: Supplementary file 2 — Additional file 2: Figure S2. Safety evaluation of MSC-ex in health mice. A. Haematoxylin and eosin (HE) staining of cardiac, liver, spleen, lung, and kidney tissues in healthy mice intravenously injected with PBS or MSC-ex. Scale bars, 40 µm. B. The levels of liver function indicators (ALT, alanine aminotransferase; AST, aspartate aminotransferase), cardiac function indicators (LDH, lactate dehydrogenase), and renal function indicators UREA in sera from PBS or MSC-ex treated mice. [file 12951_2023_1942_MOESM2_ESM.tiff]

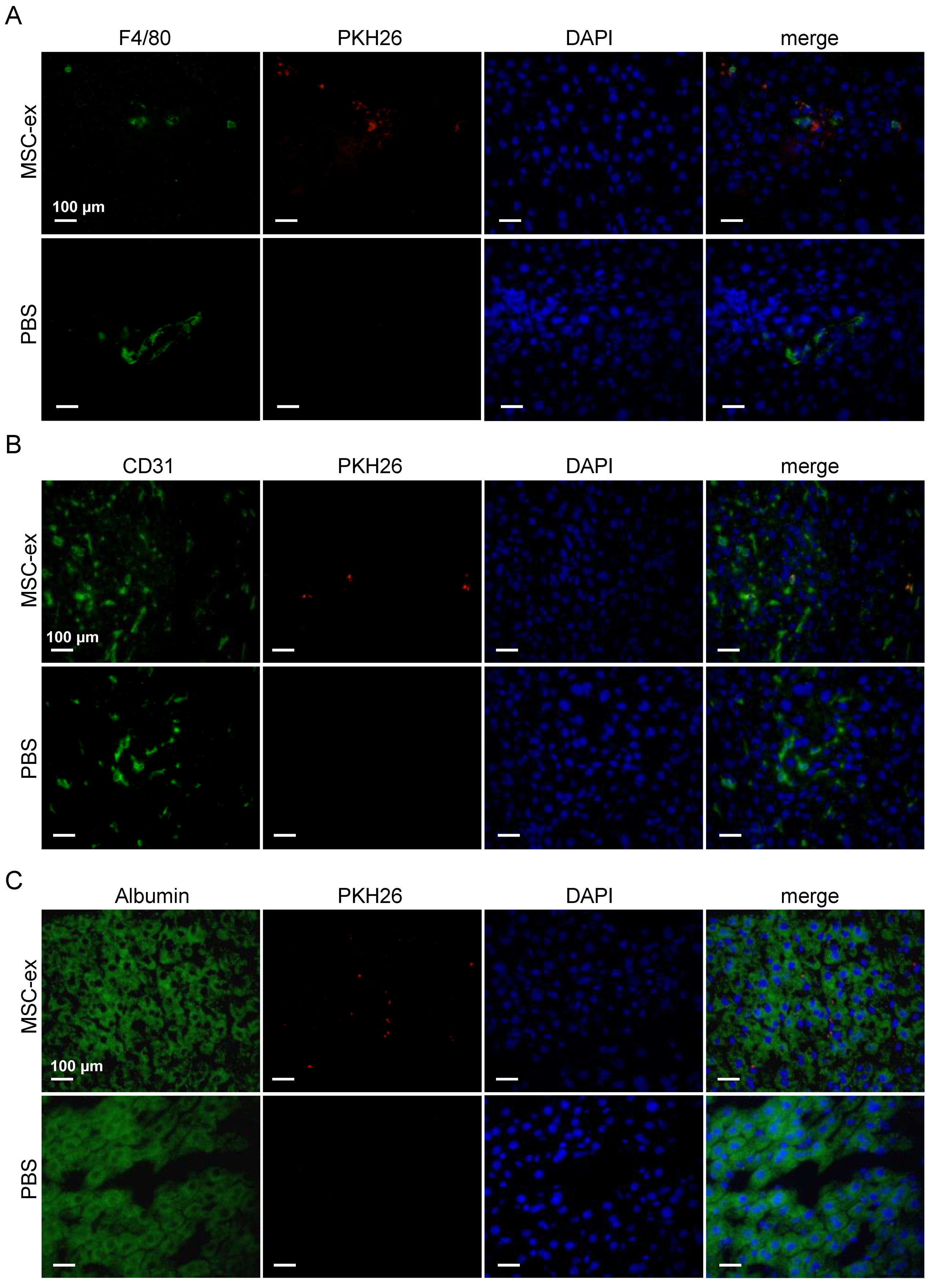

Supplement: Supplementary file 3 — Additional file 3: Figure S3. Uptake of PKH26-MSC-ex in F4/80+ Kupffer cells, albumin+ hepatocytes, or CD31+ endothelial cells in fibrotic mouse liver. A. PKH26-labeled MSC-ex was taken up by F4/80+ Kupffer cells in fibrotic mouse liver 24 h post intravenous injection. Scale bars, 100 µm. B. PKH26-labeled MSC-ex was taken up by CD31+ endothelial cells in fibrotic mouse liver. Scale bars, 100 µm. C. PKH26-labeled MSC-ex was taken up by albumin+ hepatocytes in fibrotic mouse liver. Scale bar, 100 µm. [file 12951_2023_1942_MOESM3_ESM.tiff]

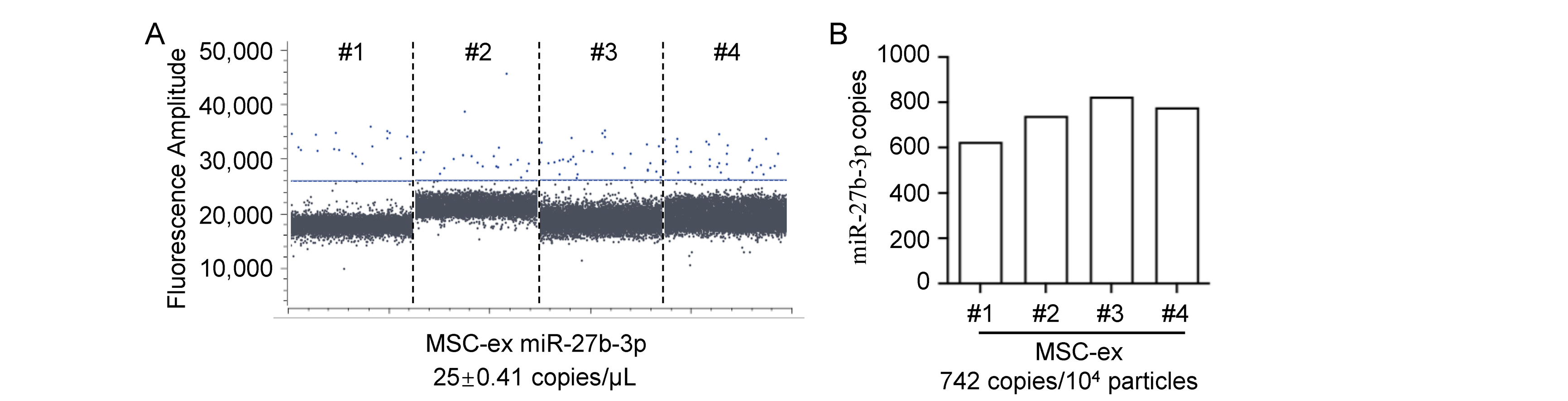

Supplement: Supplementary file 4 — Additional file 4: Figure S4. Number of miR27b-3p in MSC-ex detected by ddPCR. A. Fluorescence amplitude of miR-27b-3p in MSC-ex using ddPCR. 125 ng RNA from MSC-ex (104 particles) was used to generate cDNA by reverse transcription. ddPCR was performed using cDNA from 125 ng RNA in a total PCR system of 25 μL. The average concentration of miR-27b-3p is represented in copies/μL. B. ddPCR determined miR-27b-3p copies of MSC-ex (104 particles). Four parallel samples were tested. [file 12951_2023_1942_MOESM4_ESM.tiff]
